# Genetic diversity and population structure of *Arabidopsis thaliana* along an altitudinal gradient

**DOI:** 10.1093/aobpla/plv145

**Published:** 2015-12-15

**Authors:** Antariksh Tyagi, Shivani Singh, Parneeta Mishra, Akanksha Singh, Abhinandan Mani Tripathi, Satya Narayan Jena, Sribash Roy

**Affiliations:** 1 Genetics and Molecular Biology Division, CSIR-National Botanical Research Institute, Lucknow 226001, Uttar Pradesh, India; 2 Academy of Scientific and Innovative Research (AcSIR), Anusandhan Bhawan, 2 Rafi Marg, New Delhi 110 001, India; 3 Present address: CSIR-National Botanical Research Institute, Rana Pratap Marg, Lucknow 226001, India

**Keywords:** Chloroplast loci, evolution, genetic diversity, Indian *Arabidopsis*, microsatellite markers, phylogeography, population structure

## Abstract

The natural genetic variation within a plant species is primarily a consequence of its phylogeography and evolutionary history. This variation largely determines its present-day population structure. *Arabidopsis thaliana*, as a model plant, has been studied in great detail including its probable origin, local as well as global genetic diversity pattern, population structure, adaptation, etc. However, no such studies have so far been reported from the Indian Himalayan region. Here, we describe a comprehensive study on the genetic diversity and population structure of *A. thaliana* from an altitudinal range of 700–3400 m above mean sea level the highest altitudinal range reported so far. We also compare these populations with previously reported worldwide populations. A total of 48 accessions representing six populations were analysed using 19 microsatellites and 11 chloroplast markers. Genetic diversity analysis indicated populations to be highly diverse and comparable with worldwide populations. STRUCTURE, principal coordinate and isolation by distance (IBD) analyses showed that genetic variation in different populations is structured at geographical and altitudinal level. Further analyses indicate that these populations are genetically distinct from the rest of the world populations. Different parameters of the demographic expansion model support a rapid expansion. Based on mismatch distribution, the initial time of expansion of west Himalayan populations was found to be about 130 000 years. Bayesian analysis of divergence time indicated that these populations have a long evolutionary history in this region. Based on the results of genetic diversity parameters, demographic expansion and divergence time estimation, it appears that west Himalayan populations may be the source of the west–east expansion model.

## Abstract (precis)

In this study Tyagi et al. determined the genetic diversity and population structure of the model plant, *Arabidopsis thaliana.* These populations inhabit west Himalaya, an undersampled region. Using 19 genomic SSR and 11 chloroplast markers they determined that these populations are highly structured and genetically distinct from the rest of the world populations. They also observed that the populations were structured at the altitudinal level. Additionally their molecular clock analysis showed that these populations are not recent introductions and have inhabited the Himalayan region for about 130,000 years.

## Introduction

An understanding of the amount and distribution of genetic variability and structure in natural populations is fundamental in ecological genetic studies. The amount of genetic variability found within and among populations will both influence the response to, and be influenced by, selective pressures and evolutionary processes such as gene flow and genetic drift (Hamrick and Godt 1996; Futuyama 1998), whereas the studies on population structure can be useful for the understanding of population dynamics, historical events, etc., which in turn are greatly influenced by environmental conditions. Variations in environmental conditions may lead to phenotypic (Gugerli 1998; Till-Bottraud and Gaudeul 2002; Pluess and Stöcklin 2005; Haider *et al*. 2012) as well as genetic differentiation among populations (Ohsawa and Ide 2008).

*Arabidopsis thaliana*, commonly known as rock cress, is an annual weed having a worldwide geographical distribution. It has been extensively employed as a model plant not only for plant molecular genetics but also for ecological and evolutionary studies (Mitchell-Olds 2001; Pigliucci 2002; Weigel 2012). Thus, it is important to quantify the genetic diversity and the distribution pattern across its geographical range to understand the demographic and ecological effects (Mitchell-Olds and Schmitt 2006). There are several studies on natural genetic variation of *A. thaliana* on a regional scale in the regions of its native distribution such as Europe (Le Corre 2005; Stenøien *et al*. 2005; Bakker *et al*. 2006; Pico *et al*. 2008), Central Asia (Schmid *et al*. 2006), China (He *et al*. 2007) and Africa (Brennan *et al*. 2014), as well as in regions of presumed recent introduction and expansion such as Japan (Todokoro *et al*. 1995) and North America (Jørgensen and Mauricio 2004; Bakker *et al*. 2006). These studies have provided an insight into the genetic diversity and population structure of *A. thaliana* at a regional scale.

Being a weed, *A. thaliana* is frequently found in human-disturbed areas such as roadsides, agricultural fields, mountain slopes, etc. Seeds of *A. thaliana* are tiny and prone to transportation by humans. This artificial and unnoticed seed dispersal can disturb its phylogeographic structure. While earlier studies indicated that the present worldwide distribution of *A. thaliana* populations is unstructured or highly disturbed (King *et al*. 1993; Miyashita *et al*. 1999; Vander Zwan *et al*. 2000; Maloof *et al*. 2001), more recent studies have observed global population structure (Nordborg *et al*. 2005; Beck *et al*. 2008) in *A. thaliana*. Due to its highly self-fertilizing nature, few heritable variations exist within the populations of *A. thaliana*, and a majority of the genetic variations have been found among the populations (Hanfstingl *et al*. 1994; Todokoro *et al*. 1995; Bergelson *et al*. 1998; Breyne *et al*. 1999; Miyashita *et al*. 1999). There are also contradictory reports on correlation between geographical origin and the level of genetic variability in *A. thaliana* populations. Some studies did not find a correlation between geographical origin and genetic variability (Todokoro *et al*. 1995; Bergelson *et al*. 1998; Miyashita *et al*. 1999; Provan and Campanella 2003; Bakker *et al*. 2006; Ostrowski *et al*. 2006), while others have indicated a significant correlation (Sharbel *et al*. 2000; Schmuths *et al*. 2004; Schmid *et al*. 2006; Beck *et al*. 2008).

The origin and the biogeography of *A. thaliana* has been extensively described in several studies (Sharbel *et al*. 2000; Symonds and Lloyd 2003; Schmuths *et al*. 2004; Nordborg *et al*. 2005; Bakker *et al*. 2006; Jakobsson *et al*. 2006; Ostrowski *et al*. 2006; Schmid *et al*. 2006; Beck *et al*. 2008). However, none of these studies, including the RegMap panel study proposed by Horton *et al*. (2012), included the populations from India. This partial sampling has consistently limited the insight into *A. thaliana* population genetics, and a call for expanded sampling has been echoed by many (Hoffmann 2002; Bakker *et al*. 2006; Mitchell-Olds and Schmitt 2006; Ostrowski *et al*. 2006).

In India, *A. thaliana* is primarily found in the western Himalayas (Hooker 1875; Hoffmann 2002) with its specimen preserved in some of the herbaria in India. This region is one of the biodiversity hot spots and includes several climatic zones (Joshi and Joshi 2011). However, no detailed studies on this model species have been conducted from this region. There are only two ecotypes in *Arabidopsis* stock Centre (ABRC), which have been reported from this region, *viz.* Kas-1 and Kas-2, collected from Kashmir region of India, but concerns have been raised about the origin of Kas-1 accession, as it stands aloof from the rest of Asian accessions in phylogenetic studies (Yin *et al*. 2010). Additionally, its genomic signatures show higher affinity towards European accessions, and it is presumed that Kas-1 was transported from Europe to India sometime in the 19th century (Vander Zwan *et al*. 2000). In a recent report, 77 accessions from Asia, Europe, North Africa, North America and Atlantic islands using 11 chloroplast (CP) markers were studied (Yin *et al*. 2010). Their study suggested that the extant populations along the Yangtze River might have dispersed there via the west Himalaya or Kunlun mountains during postglacial expansion and underwent rapid expansion around 90 000 years ago. Conversely, they have further suggested that Yangtze River populations might be the source of present Himalayan populations. Inclusion of populations from west Himalaya might shed light in this direction.

In the present study, our goals were (i) to estimate the genetic diversity of *A. thaliana* populations from the west Himalayan region, which had never before been analysed, (ii) to establish the relationship of *A. thaliana* of west Himalaya with respect to other accessions of the world and (iii) to determine the probable origin of west Himalayan populations and their expansion. We attempt to provide here the link between populations of China and west Himalaya and further extend the demographic model proposed by Sharbel *et al*. (2000), where the authors suggested that the central Asian and Peninsula region might be the source of European colonization. Our sample sets include 48 accessions from six populations and were analysed using nuclear microsatellite (MS) and CP loci. These populations were further compared with worldwide accessions reported by Yin *et al*. (2010). Recently, our group has characterized four of these populations using morphological traits and has shown that some of the morphological traits were highly correlated with their corresponding altitudes (Singh *et al*. 2015). Inclusion of Indian populations of *A. thaliana* will greatly enhance our understanding of global population structure and evolution of this model species.

## Methods

### Collection of accessions

**Figure 1.**
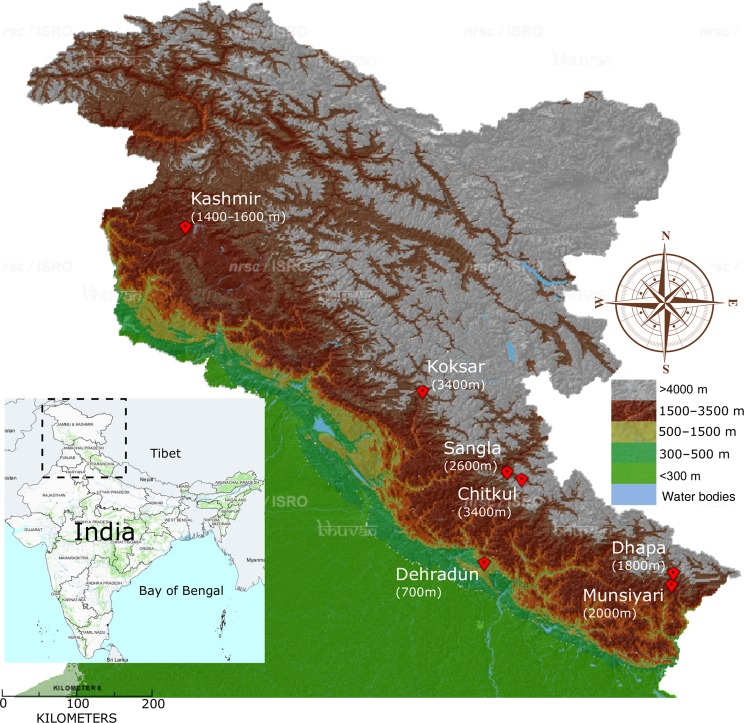
Geographical locations of sample collection sites. Terrain map of west Himalaya showing sample collection sites (red diamonds). Figures in parentheses indicate site altitude in a.m.s.l. Inset: political map of India. Image courtesy: NRSC/ISRO Bhuvan, India.

A total of 48 *A. thaliana* accessions were collected from six different populations located at Dehradun [Deh, 700 m above mean sea level (a.m.s.l.)] designated as low altitude; Dhapa (Dha, 1800 m a.m.s.l.) and Munsiyari (Mun, 2000 m a.m.s.l.), designated as medium altitude and Sangla (San, 2600 m a.m.s.l.), Koksar (Kok, 3400 m a.m.s.l.) and Chitkul (Chi, 3400 m a.m.s.l.), designated as high-altitude localities of the west Himalayan region (Fig. 1). No specific permissions were required for sample collections from these locations as these do not come under any protected area. Further, *A. thaliana* is not an endangered or protected species. Each population was geo-referenced for their latitude, longitude and altitude with a global positioning system receiver (Garmin, USA). The minimum and maximum distances between populations were 4 km (Mun and Dha) and 384 km (Kok and Mun), respectively. The accessions were named by the first three letters of the name of the nearest location or village from where samples were collected, followed by a numeric indicating the individual accession number. The habitats of the populations varied from human-disturbed lawn to undisturbed mountainous river valley and slopes **[see** **Supporting Information—Table S1****]**.

### DNA isolation, sequencing and genotyping

Genomic DNA was isolated from field-collected leaf samples using the Qiagen™ DNeasy Plant mini kit (USA) following the manufacturer's protocol. The quality of the DNA was checked on 0.8 % agarose gel and quantified using Nanodrop spectrophotometer (Thermofisher, USA). For amplification of CP loci, the same set of primer pairs was used as described in Yin *et al*. (2010). Polymerase chain reaction (PCR) was performed in 20 µL reaction volume containing 10 µL of 2× Amplitaq Gold 360 Master Mix (Applied Biosystems, USA), 40–50 ng template DNA and 10 pmol of each of the forward and reverse primers. The thermocycler programme was an initial denaturation at 94 °C for 4 min followed by 35 cycles of denaturation at 94 °C for 30 s, annealing at 50–59 °C for 30 s (depending on *T*_m_ of primer pairs) and extension at 72 °C for 1 min followed by a final extension step at 72 °C for 5 min. The amplified products were cleaned up using the Qiaquick PCR purification kit (Qiagen, USA). Sequencing PCR was performed using ABI Prism BigDye Terminator v3.1 cycle sequencing Kits (Applied Biosystems) in 10 µL reaction volume containing 2 µL cleaned PCR product as template, 1 µL of either forward or reverse primer, 0.5 µL of RRmix and 1.75 µL of 5× dilution buffer. The products were ethanol precipitated and denatured with Hi-Di formamide and sequenced on ABI 3730XL Automated DNA Sequencer (Applied Biosystems).

All the samples were genotyped for 19 previously described MS loci (Bell and Ecker 1994) through fluorescent labelling PCR using M13 universal primer following Schuelke (2000). An 18-bp tail (5′-TGTAAAACGACGGCCAGT-3′) complementary to M13 sequence was added to each forward MS primer. Four different fluorescent dyes were used to label the M13 primer: 6-FAM (blue), NED (yellow), PET (red) and VIC (green). Each MS marker was optimized for annealing temperature. Each 20 µL reaction contained 2.5 mm MgCl_2_, 0.2 mm deoxyribonucleotides, 4 pmol fluorescently labelled M13 primer, 4 pmol of forward primer with M13 tail and 8 pmol of MS reverse primer, 2 µL Promega buffer and 0.5 U of Promega *Taq* polymerase. The PCR reactions were performed in the following conditions: 94 °C (1 min), then 30 cycles at 94 °C (1 min)/specific annealing temperature (56–60 °C) for each MS marker, (1 min)/72 °C (1 min), followed by 8 cycles at 94 °C (1 min)/53 °C (1 min)/72 °C (1 min) and a final extension at 72 °C for 10 min. The amplified products were run on a capillary-based 3730xl DNA Analyser (Applied Biosystems) and the products were precisely sized for major, comparable and conspicuous peaks using GeneMapper v4.0 (Applied Biosystems), using default quality indicators of GeneMapper. Details of the MS data are shown in **Supporting Information—Table S2**.

### Sequence alignment

We sequenced 11 CP loci from 8 individual plants collected from each of the 6 locations (total 528 sequences). Each sequence was visually inspected and matched with its corresponding electropherogram. Only the high-quality sequences (Phred score >20) were considered for analysis. Besides these 528 sequences, we included the sequences of worldwide samples provided in for comparative analyses. The average sequence length of our samples was slightly shorter than the sequence length obtained by Yin *et al*. (2010). Therefore, the sequences were trimmed individually from both sides to avoid large gaps in the aligned data set. DNA sequences were aligned using MUSCLE (Edgar 2004) implemented in MEGA 5 (Tamura *et al*. 2011). After alignment, the sequences were edited ‘by eye’ to minimize any misalignments or erroneous introduction of indels. The sequences were concatenated and the resulting aligned length of the sequences was 6299 nucleotides.

### Population data analysis

Three sets of population data were considered for analysis: (i) 48 accessions from six different populations from west Himalaya using 19 MS markers, (ii) 48 west Himalayan accessions using 11 CP loci and (iii) 48 west Himalayan accessions and 77 worldwide accessions using 11 CP loci from Yin *et al*. (2010). While the data sets (i) and (ii) were used for genetic analyses of only Indian populations, data set (iii) compared west Himalayan and the rest of the world populations. Population genetic parameters, *viz.* genetic diversity measures, such as the percentage of polymorphic loci (%PL), mean number of alleles (Na), mean number of effective alleles (Ne), mean allelic richness (Rs), mean private allelic richness (Rp), mean gene diversity (Hs), number of multilocus haplotypes (Nh), expected heterozygosity (He) and observed heterozygosity (Ho) were estimated for the three data sets using software programs GENALEX 6.5 (Peakall and Smouse 2012), FSTATv 2.9.3 (Goudet 1995) and HP-Rare v.1.0 (Kalinowski 2005). Allelic richness and private allelic richness were estimated to the smallest sample size (*n* = 2) using HP-rarefaction tools to avoid sampling disparity.

Genetic differentiation among populations was estimated by analysis of molecular variance (AMOVA) using Arlequin v3.5 (Excoffier and Lischer 2010), which calculates *F*_ST_ like statistics. Statistical significance of AMOVA was tested from 20 000 permutations. To determine whether the genetic distances between populations were determined by geographic distance or difference in their altitude, IBD analysis between populations was performed. To determine the IBD, partial Mantel test (PMT) were performed. This test allows two pairwise distance matrices to be compared, while controlling for the effect of a third. Partial Mantel test was performed between pairwise *F*_ST_ and the corresponding paired population matrix of geographic distances while controlling for pairwise altitudinal distances. Alternatively PMTs were performed between pairwise *F*_ST_ and pairwise altitudinal distances while controlling for geographic distances. Pairwise *F*_ST_ between six populations were calculated using the Arlequin program. Geographic distances between location pairs were calculated using Geographic Distance Matrix Generator v1.2.3 software (Ersts 2011). Partial Mantel tests were performed with 999 permutations using the PASSaGE program (Rosenberg and Anderson 2011). To determine whether the within-population genetic diversity was correlated with altitudes, Pearson's correlation test was performed between %PL and corresponding altitudes of the sites.

The population structure and assignment of individual west Himalayan accessions to populations on the basis of MS genotype data were performed using STRUCTURE v2.3.3 (Pritchard *et al*. 2000) and principal coordinate analysis (PCoA). In STRUCTURE, model settings included diploid multilocus genotypes, correlated allele frequencies between populations, admixture model and running the algorithm with 200 000 burn-in MCMC iterations and 500 000 MCMC post burn-in repetitions for parameter estimation. To determine the appropriate value of *K*, STRUCTURE was run in 20 replicates with assumed *K* from 2 to 10. The *K* value where the posterior probability (ln *P*(*D*)) began to plateau was selected as the true *K* (Wang *et al*. 2013). When varied from 2 to 10, the ln *P*(*D*) began to plateau at *K* = 6 **[see** **Supporting Information—Fig. S1****]**, which was also incidentally the number of our populations. To determine how the individuals of populations respond to increasing *K*, we plotted STRUCTURE results from *K* = 2 to *K* = 6. Principal coordinate analysis was performed using Nei's distance matrix as implemented in GENALEX 6.5, and the resulting eigenvalues were plotted using *k*-means clustering implemented in cluster package R v3.2 (http://www.R-project.org). Phylogenetic relationships among accessions were determined by the neighbour-joining (NJ) method using PAUP*4.0 (Swofford 2003). Visual observation showed the presence of an insertion, TTCTAAT, at position 245 in Himalayan and Yangtze River accessions, TTCTA at position 465 and TGGAT at position 1528 in the rest of the world accessions in the concatenated sequence data set. Therefore, we decided to include indels in the analysis. It has been suggested that inclusion of indels in phylogenetic analysis is a reasonable choice to avoid loss of important character states and has been used for CP sequences to resolve branching order, phylogenetic resolution and nodal branch support (Crayn and Quinn 2000; Ingvarsson *et al*. 2003; Egan and Crandall 2008). The indels were coded using the modified complex indel coding (MCIC) method (Müller 2006) using SeqState version 1.4.1 (Müller 2005). The NJ analysis was performed with 1000 bootstrap replicates.

Haplotype diversity in the combined sequence data was determined using DnaSP v5 (Rozas *et al*. 2003). Haplotype network was generated using TCS v1.21 (Clement *et al*. 2000) implemented in PopART (http://popart.otago.ac.nz). The haplotype with the highest out group weight as estimated by TCS was considered as the ancestral haplotype.

The more intensive sampling of west Himalayan populations can cause sampling bias when comparing with the rest of the world populations. Therefore, one accession per population was randomly selected for phylogenetic and haplotype analyses.

For MS loci, pairwise Nei's genetic distances were determined using GENALEX 6.5 and the matrix was used to construct majority rule consensus tree of 500 unrooted NJ trees using PHYLIP package (Felsenstein 2005).

### Demographic expansion and estimation of divergence time

To test the neutral mutation hypothesis, different statistical tests such as Tajima's *D* (Tajima 1989) and Fu and Li's D*, *D*, F* and *F* test (Fu and Li 1993) were conducted for each marker using DnaSP v5 (Rozas *et al*. 2003) with *A. arenosa* as out group (Yin *et al*. 2010). Chloroplast markers are reported to be reliable indicators of demographic events due to their lack of ability to recombine (Beck *et al*. 2008). Therefore, we chose CP markers to determine the mismatch distribution using ARLEQUIN. The ‘raggedness statistic’ (Rag), mode of mismatch distribution (*τ*) and ‘sum of squared deviation’ (SSD) values were determined to infer the demographic expansion event. To estimate the time since expansion, substitution rate of 2.9 × 10^−9^ changes per site per year for non-coding sequence of the *A. thaliana* CP was considered as reported by Sall *et al*. (2003). For estimation of time since expansion, the following equation was adopted:

$$t=\frac{\tau }{2\mu }$$

where *t* is the expansion time in number of generations, *τ* is the mode of the mismatch distribution and *μ* is the mutation rate per generation for the entire DNA sequence.

The divergence time between the accessions was estimated using Bayesian methods implemented in BEAST v1.8.0 (Drummond *et al*. 2012). One accession per west Himalayan population was used for analysis as described above. Three TMRCA (Time to Most Recent Common Ancestor) reference points were used to calibrate the clock and estimate the divergence time in rooted time-measured molecular phylogenies of the concatenated data set: First, 11.0–14.0 million years ago (mya) divergence time between *Olimarabidopsis cabulica* and *A. thaliana* (Koch *et al*. 2001); second, 12 000–200 000 years divergence time between the allotetraploid *A. suecica* and its parent *A. thaliana* (Jakobsson *et al*. 2006) and third, 5.0 mya divergence time between *A. thaliana* and *A. arenosa* (Koch *et al*. 2000; Yin *et al*. 2010). The TMRCA reference point between *A. suecica* and *A. arenosa* was not used as *A. arenosa* is the paternal parent of *A. suecica* and all the sequences in our data set are of CP origin. We excluded Kas-1 (IND) and XJalt, XJqhx (CHN) from their parent data set and included in the rest of the world data set, as the phylogenetics and haplotype network analyses grouped these accessions with the rest of the world (see Results). Four independent BEAST runs of 3 × 10^7^ generations saving logs every 200 generations were performed. Results of four BEAST runs were combined and then subsampled at a lower frequency using Log Combiner v1.8.0; this produced 3 × 10^4^ age estimates under the coalescent constant size tree prior and the uncorrelated lognormal relaxed clock. The count of beast runs was decided to achieve an effective sample size of at least 100 for all TMRCA estimates. The MCMC data output for each individual run was visualized using Tracer v1.5. Chronogram trees were generated using TreeAnnotator v1.8.0 program.

## Results

### Genetic diversity and structure of west Himalayan populations of *A. thaliana*

Genetic diversity of the west Himalayan populations was estimated using 19 MS and 11 CP loci. Among the 19 MS markers, no single marker was found to be monomorphic **[see** **Supporting Information—Table S2****]**. The various measures of gene diversity parameters for the overall population are depicted in Table 1, and population-level diversity parameters for MS and CP markers are depicted in Tables 2 and 3, respectively. The data indicated that San and Chi were the most and the least diverse populations, respectively (Table 2). The amount of genetic variation in terms of %PL and allelic richness per population did not exhibit any significant correlation with their corresponding altitudes (*r* = 0.06, *P* = 0.905). All the 11 CP loci were found to be polymorphic. At population level, Deh and Chi populations were found to be the most and the least diverse with respect to %PL and Na using CP markers. The He (assuming panmictic populations) was higher than the Ho. The haplotype diversity of each of the six populations was 1.0 indicating high degree of CP sequence diversity across all the populations (Table 2).

**Table 1.** Genetic diversity of *A. thaliana* from west Himalaya and the rest of the world. CP, chloroplast loci; MS, microsatellite markers; RW, rest of the world; YR, Yangtze River; WH, west Himalaya; *n*, number of samples; PL, polymorphic loci; Na, number of alleles; Ne, number of effective alleles; Nh, number of haplotypes; Hd, haplotype diversity; Rs, allelic richness; Rp, private allelic richness and Hs, gene diversity. Na, Ne, Hd, Rs, Rp and Hs values are mean ± SD estimated from 11 CP loci and 19 MS markers for WH samples.

Marker set	Population	*n*	%PL	Na	Ne	Nh	Hd	Rs	Rp	Hs
CP	RW	59	69.8	1.708 ± 0.018	0.196 ± 0.008	37	0.9731	–	–	–
	YR	17	28.12	1.283 ± 0.018	0.127 ± 0.009	4	0.5956	–	–	–
	WH	49	62.6	1.636 ± 0.019	0.206 ± 0.008	48	1	–	–	–
MS	WH	48	100	6.0 ± 1.14	2.907 ± 0.443	–	1	3.74	3.59	0.628 ± 0.131

**Table 2.** Genetic diversity of *A. thaliana* populations from west Himalaya using MS loci. *n*, mean number of accessions per MS loci; PL, polymorphic loci; Na, number of observed alleles; Ne, number of effective alleles; He, expected heterozygosity; Ho, observed heterozygosity; Rs, allelic richness and Rp, private allelic richness. All values are mean ± SD estimated from 19 MS markers.

Pop (altitude a.m.s.l.)	*n*	%PL*	Na*	Ne*	He	Ho	Rs	Rp
Chi (3400)	7.26 ± 0.12	57.89	2.05 ± 1.12	1.63 ± 0.86	0.25 ± 0.04	0.07 ± 0.1	1.99 ± 1.07	0.38 ± 0.82
Deh (600)	7.47 ± 0.11	68.4	2.42 ± 1.67	1.76 ± 0.97	0.31 ± 0.06	0.08 ± 0.14	2.31 ± 1.44	0.64 ± 0.92
Dha (1800)	7.47 ± 0.15	68.42	2.26 ± 1.09	1.76 ± 0.72	0.29 ± 0.05	0.11 ± 0.23	2.23 ± 1.05	0.42 ± 0.49
Kok (3400)	7.36 ± 0.13	78.95	2.36 ± 1.21	1.85 ± 0.91	0.39 ± 0.04	0.07 ± 0.16	2.33 ± 1.14	0.48 ± 0.81
Mun (2000)	7.31 ± 0.15	78.95	2.21 ± 0.85	1.59 ± 0.61	0.33 ± 0.06	0.1 ± 0.18	2.15 ± 0.84	0.39 ± 0.76
San (2600)	7.36 ± 0.15	94.6	3.10 ± 1.04	1.87 ± 0.70	0.36 ± 0.05	0.13 ± 0.08	2.91 ± 0.96	1.21 ± 0.76

**Table 3.** Genetic diversity of *A. thaliana* populations from west Himalaya using CP loci. *n*, number of samples; PL, polymorphic loci; Na, number of observed alleles; Ne, number of effective alleles and Hd, haplotype diversity. All values are mean ± SD.

Population	*n*	%PL	Na	Ne	Hd
Chi	8	5.12	1.051 ± 0.012	1.02 ± 0.004	1
Deh	8	54.16	1.542 ± 0.011	1.411 ± 0.003	1
Dha	8	16.20	1.164 ± 0.011	1.052 ± 0.002	1
Kok	8	25.16	1.254 ± 0.013	1.171 ± 0.003	1
Mun	8	7.04	1.07 ± 0.010	1.031 ± 0.003	1
San	8	10.66	1.107 ± 0.016	1.056 ± 0.006	1

**Figure 2.**
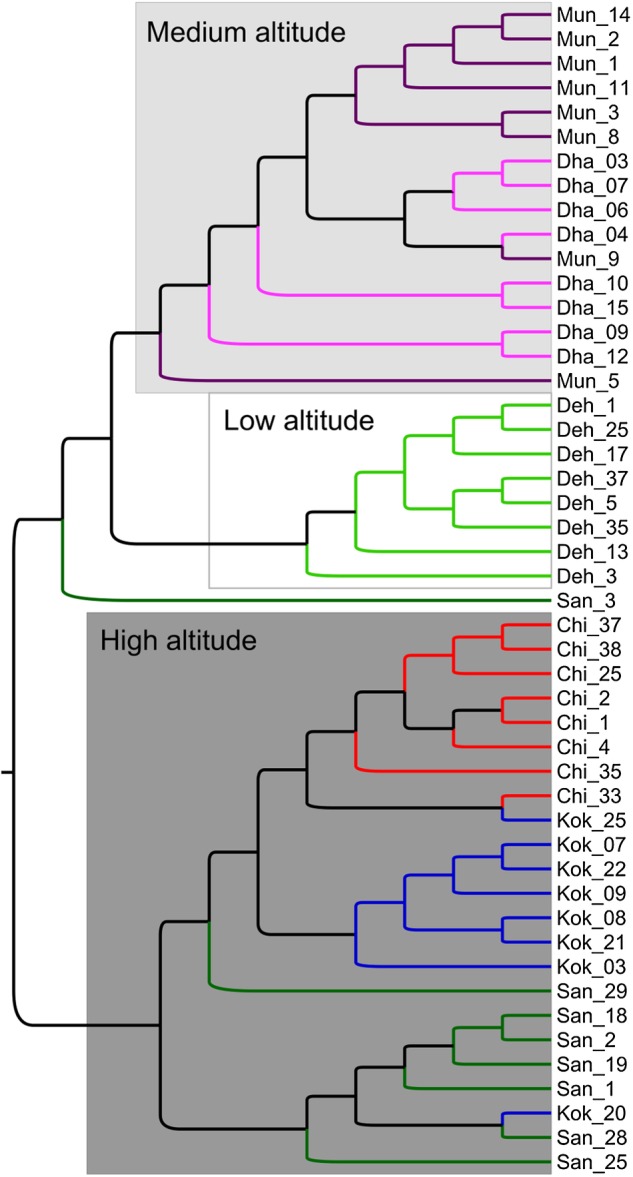
Majority rule consensus tree of 500 un-rooted NJ trees. Genetic distance matrix of 19 MS markers was used for construction of the tree. The three clades corresponding to the populations from the three altitudinal zones (low, medium and high) are shown as shades of grey. All the branches are 100 % supported.

In the phylogenetic analysis using NJ method with MS markers, clustering of accessions at two hierarchical levels was observed. The three major branches represented accessions on the basis of their defined altitudinal class, whereas branches arising from external nodes formed clades representing accessions clustered according to population affiliation, except Mun and Dha that clustered under a single clade. All the other accessions clustered according to their population affiliation with minor intermixing (Fig. 2). Neighbour joining tree of CP loci did not show such grouping observed with MS marker analyses **[see** **Supporting Information—Fig. S2****]**. In this tree, there were two distinct clusters with high bootstrap support; Cluster I consisted of all the accessions except for Kok. The accessions in Cluster I formed a highly polytomous branch as only 0.9 % of the nucleotide sites were parsimony informative in the aligned data set out of which 38 % were contributed by Kok. Analysis of molecular variance estimates of genetic differentiation using MS and CP loci exhibited 49.5 and 43.5 % of the genetic variation, respectively, among the populations. However, the average *F*_ST_ values estimated from both the marker sets were significant (*F*_ST_ = 0.495 and 0.435, respectively; *P* < 0.0001). On the basis of MS data, all the six populations differed genetically from each other since all the *F*_ST_ values between pairs of populations were significant (*F*_ST_ = 0.18993–0.61515; *P* < 0.0001) **[see** **Supporting Information—Table S3****]**. Partial Mantel tests of pairwise geographic distances **[see** **Supporting Information—Table S4****]** and corresponding *F*_ST_ values (while controlling for pairwise altitudinal differences) showed a significant correlation (*r*^2^ = 0.57, *P* = 0.028) indicating IBD, whereas no significant correlation was found when pairwise *F*_ST_ were correlated with pairwise altitudinal differences (*r*^2^ = 0.54, *P* = 0.07).

**Figure 3.**
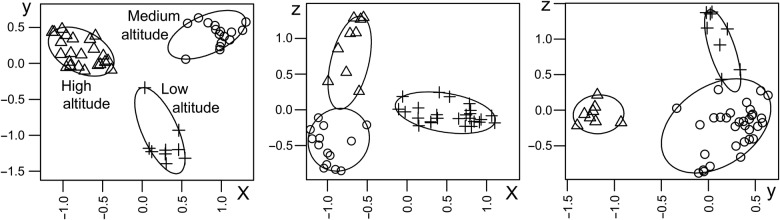
*K*-means clustering (circled) of first three coordinates from principal co-ordinate analysis of the MS markers. Accessions from low, medium and high altitude formed different clusters. The first three co-ordinates (*x*-axis, first co-ordinate; *y*-axis, second co-ordinate; *z*-axis, third co-ordinate) are shown.

**Figure 4.**
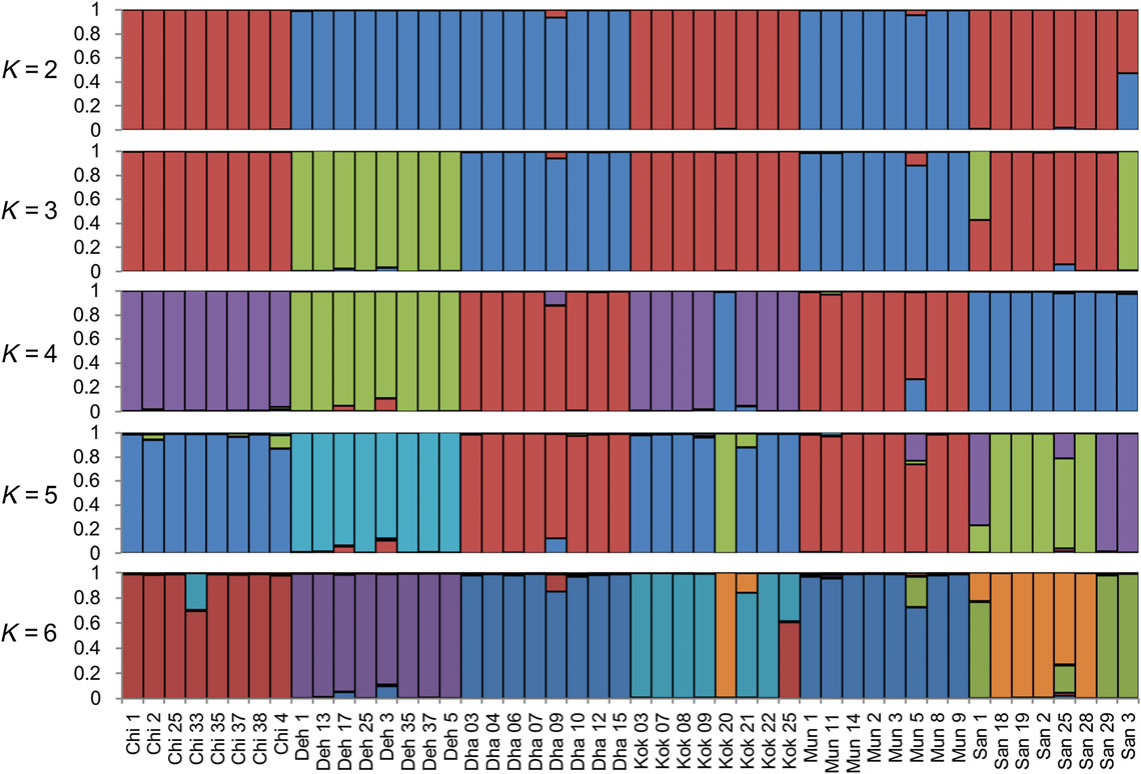
Genetic group assignment of west Himalayan accessions based on majority membership proportion inferred from *K* = 2 to *K*= 6 using STRUCTURE program. Each accession is represented by a single vertical line broken into *K* coloured segments, with lengths proportional to each of the *K* inferred clusters.

To further assess the genetic structure, populations were analysed using two clustering approaches, PCoA and STRUCTURE. Principal coordinate analyses of the MS markers explaining 19.02, 9.98 and 9.03 % of the overall genetic variation identified three groups representing three altitudinal classes (Fig. 3; **Supporting Information—Fig. S3**). STRUCTURE analysis indicated that at *K* = 2, the populations were grouped into two: one representing populations of low and medium altitude, and the other one representing populations of high altitude. At *K* = 3, all the populations grouped according to their altitudinal assignment (described in Methods). Further, at *K* = 4 and 5, San formed a separate group from Chi and Kok, and at *K* = 6, all the populations except Mun and Dha formed separate groups. San showed large within-population variations at *K* = 5 and 6 (Fig. 4). The accessions that were highly variable in STRUCTURE analysis were intermixed in NJ analysis as well. For example, Kok-20, San-29 and San-3 were found to be highly variable in both the analyses.

### Comparison between west Himalayan and the rest of the world populations

The west Himalayan populations were compared with the Yangtze River and the rest of the world populations using CP sequences (data set iii). The results of comparative genetic diversity parameters between the three groups are depicted in Table 1. West Himalaya and the rest of the world showed high and comparable genetic diversity within populations. Thirty-seven haplotypes were detected from 75 accessions of the world data set, whereas 48 haplotypes were identified from the 49 accessions of west Himalaya. Yangtze River populations could be defined by four haplotypes. Haplotype diversity of the west Himalayan population was slightly higher than world populations (Table 1), whereas Yangtze River populations had the smallest haplotype diversity.

**Figure 5.**
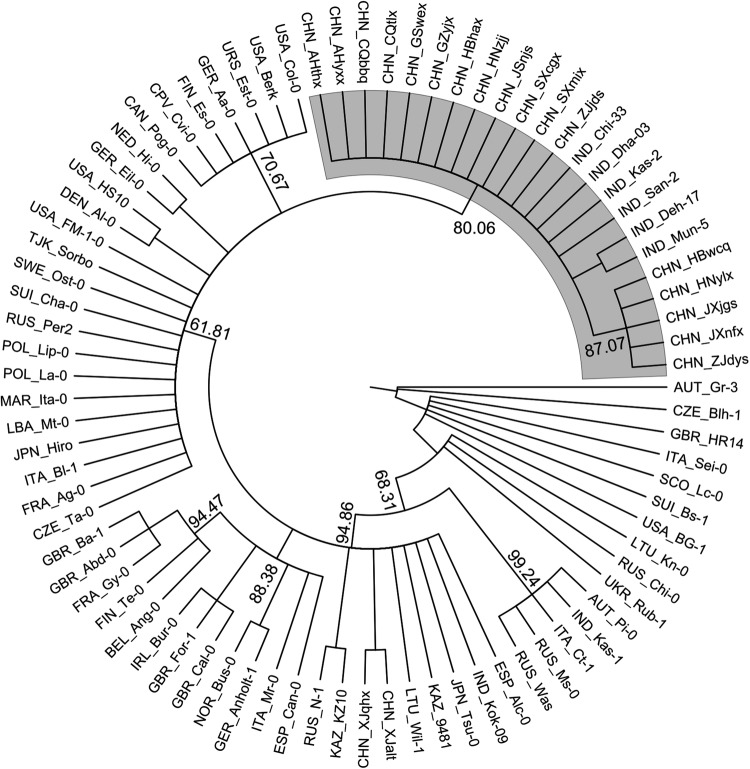
Neighbour-joining bootstrap consensus tree of west Himalaya and the rest of the world accessions. The tree was constructed using the concatenated data set of 11 chloroplast markers and following the indel coding method, MCIC. Numbers at the nodes are bootstrap values as percentages of 1000 replicates. Grey-shaded clade represents Cluster I, and the rest of the accessions are part of Cluster II.

**Figure 6.**
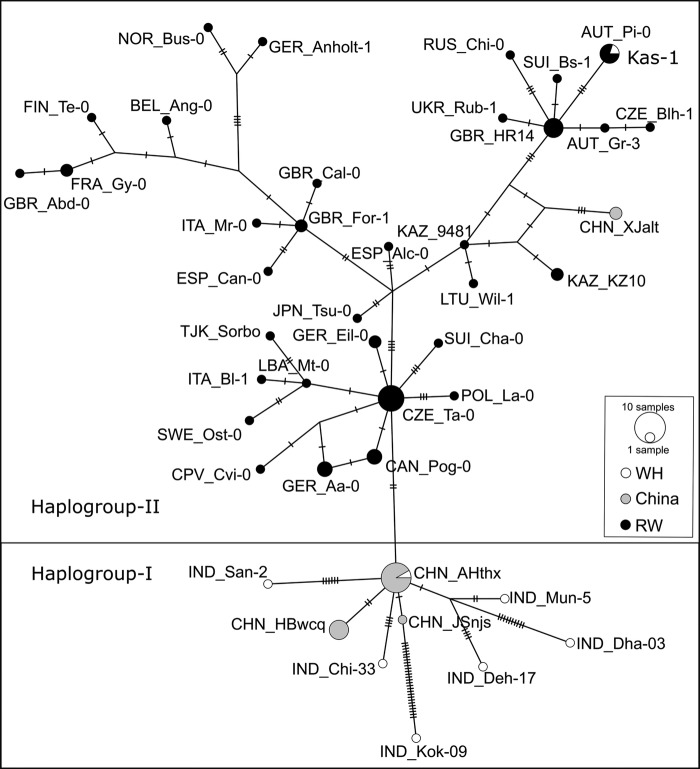
Haplotype network. There were a total of 46 haplotypes from 83 accessions from combined data set of *A. thaliana* accessions as inferred using concatenated sequences of 11 CP markers. The Haplogroup I contains the accessions from west Himalaya and Yangtze region, and the Haplogroup II contains the accessions from the rest of the world. Individual discs indicate haplotypes with the size of the disc proportional to the number of accessions in the haplotype **[see** **Supporting Information—Table S6****]**. Hatch marks denote nucleotide changes.

In the concatenated data set of 11 CP loci, 1.5 % were parsimony informative characters (PIC). The genetic relationship among west Himalayan and the rest of the world accessions was first analysed by constructing NJ trees with complete deletion of gaps. This method resulted in a poorly supported tree (data not shown). However, inclusion of indels greatly increased the statistical branch support without changing the clustering pattern as observed with complete deletion methods (Fig. 5). There were two distinct clusters with high bootstrap support: Cluster I consisted exclusively of the accessions from west Himalaya and the Yangtze River, whereas Cluster II consisted of the accessions from the rest of the world. The accessions in Cluster I formed a highly polytomous branch. The rest of the world's accessions clustered into sub-clusters without any preference to geographical affiliation. Interestingly, the accessions from Xinjiang province of China (XJalt, XJqhx) and Indian accession Kas-1 did not group with Cluster I. Further, the haplotype network was constructed with 95 % of steps connection limit. The network consisted of 46 haplotypes (Fig. 6; **Supporting Information—Table S6**). Two haplogroups (I and II) were detected, where Haplogroup I consisted of all the accessions from west Himalaya and Yangtze River, and Haplogroup II consisted of the rest of the world accessions including accessions from Xinjiang province and Kas-1. The Haplogroup I was connected to Haplogroup II by two nucleotide character states (G-266-C, *accD*; G-4138-C, *rpoB*). The ancestral haplotype analysis showed one haplotype (CHN-AHyxx) consisting of 10 accessions from the Yangtze River and one accession from west Himalaya (Kas-2) belonging to Haplogroup I, and another haplotype (CZE Ta-0) belonging to Haplogroup I, consisting of five accessions, had the highest and outgroup weights (0.11 and 0.12, respectively) among all the detected haplotypes. This indicates that these two haplotypes are the ancestral haplotypes for the respective haplogroups **[see** **Supporting Information—Table S5****]**.

### Demographic expansions and divergence time of west Himalayan populations based on CP loci

Tajima's *D* values for all the CP loci were found to be negative and mostly significant (in 7 out of 11 markers) at *P* < 0.05. Tajima's *D* value for the concatenated data set was also negative and highly significant (*D* = −2.36, *P* < 0.01). Similarly, Fu and Li's D*, *D*, F* and *F* test values were also negative for all but *trnG*. The combined values were also negative and significant at *P* < 0.01 **[see** **Supporting Information—Table S6****]**, indicating a rapidly expanding population when compared with the Yangtze River population (Tajima's *D* = −1.17) (Yin *et al*. 2010). The results of mismatch distribution analysis were also consistent with this finding **[see** **Supporting Information—Table S7****]**. The SSD and raggedness index between the observed and expected mismatch distribution in west Himalayan (SSD = 0.1, *P* = 0.6, Rag = 0.004, *P* = 0.43) were smaller than that of Yangtze River (SSD = 0.15, *P* = 0.9, Rag = 0.57, *P* = 0.04). These results provided evidence of rapid population expansion in the west Himalayan population. Based on the *τ* value, the initial time of expansion of west Himalayan populations and Yangtze River populations was found to be ∼130 000 and ∼84 000 years, respectively.

Using Bayesian approaches, we estimated the divergence time with 95 % highest probability density (HPD) **[see** **Supporting Information—Table S8****]**. The TMRCA of combined populations of western Himalaya and Yangtze River was estimated to be 0.45 mya (95 % HPD, 0.35–0.56), which was exactly same as the TMRCA of western Himalaya lineage. The TMRCA of Yangtze River populations stood at 0.29 (95 % HPD, 0.20–0.39). Further, the origin of the rest of the world was found to be 0.62 mya (95 % HPD, 0.49–0.74). Although the tree prior for *A. arenosa* and *A. thaliana* split was 5.0 mya, the relaxed molecular clock found it to be 6.61 mya (95 % HPD, 5.94–7.34). The rest of TMRCA reference points, *O. cabulica* (12.42 mya; 95 % HPD, 12.17–12.68) and *A. suecica* (0.64 mya; 95 % HPD, 0.51–0.76), were within the range of the given priors.

**Figure 7.**
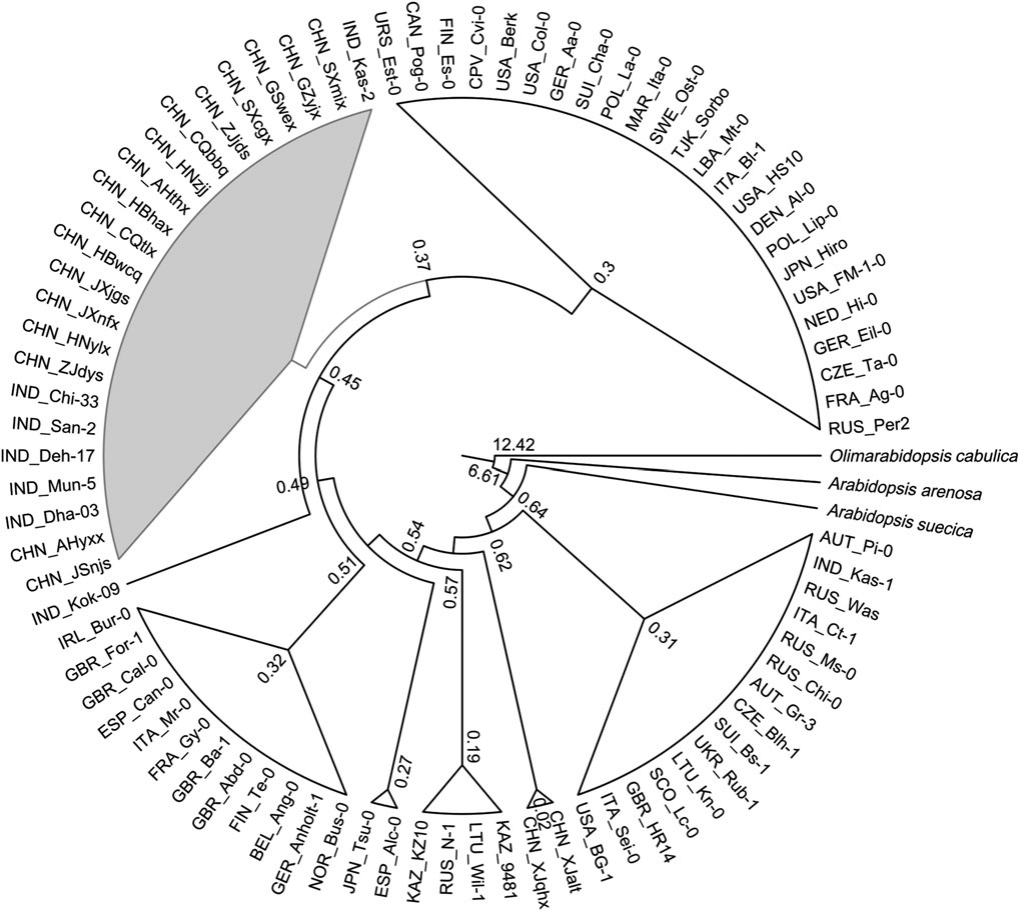
Estimation of divergence time. Chronogram generated by four independent BEAST runs of 3 × 10^7^ generations each under the coalescent constant size tree prior and an uncorrelated lognormal relaxed clock using concatenated data set of 11 CP markers. Values on the nodes indicate divergence time in million years ago. Grey-shaded clades contain accessions from west Himalaya and Yangtze River, and white clades contain accessions from the rest of the world.

The chronogram resulting from Bayesian phylogenetic time tree analysis had two distinct clades. One included accessions from west Himalaya, Yangtze River, and the other included all the accessions from the rest of the world accessions. The chronogram clearly showed a split between European and Himalaya–Yangtze River accessions at 0.49 mya (Fig. 7).

## Discussion

The Himalayan region is one of the mega-biodiversity hot spots of the world, bestowed with rich flora and fauna (Chatterjee 1939; Nayar 1996; Pandit *et al*. 2000). Despite its floristically rich heritage, *A. thaliana*, a well-studied model plant species, has found no detailed studies from this region. We collected accessions of *A. thaliana* from the west Himalaya ranging altitudes of 700–3400 m a.m.s.l. The populations were collected from diverse habitats of highly disturbed lawn (Deh) to mountainous slopes (Mun) to undisturbed mountainous river valley (Chi). To the best of our knowledge, this is the first report on *A. thaliana* populations from altitudes ranging from such a large altitudinal range.

The six west Himalayan populations analysed with MS and CP loci indicated substantial genetic variation, with all the pairwise *F*_ST_ values being significant. Most of the earlier studies have reported less genetic variation within populations, consistent with *A. thaliana* life history and more among populations (Hanfstingl *et al*. 1994; Bergelson *et al*. 1998; Breyne *et al*. 1999; Miyashita *et al*. 1999; Jørgensen and Mauricio 2004; Nordborg *et al*. 2005; Beck *et al*. 2008; Brennan *et al*. 2014). In other cases, higher genetic variation was observed within population than among populations (Jørgensen and Mauricio 2004; Stenøien *et al*. 2005; Bakker *et al*. 2006; Pico *et al*. 2008; Lundemo *et al*. 2009). These differences in genetic differentiation may arise to some extent due to use of different markers and sampling strategies (Nordborg *et al*. 2005). High variations within populations have been attributed to differential contributions of migration, outcrossing and *de novo* mutations (Pico *et al*. 2008). In the case of west Himalayan populations, owing to its high mountain ranges, migration may play a limited role in the advent of within-population variations. Only in Mun and Dha populations, for their geographical and altitudinal proximity and small *F*_ST_ value, migration might play a role in within-population variability. With the present data set, it was not possible to comment on the contribution of neutral changes to the within-population variability.

The results of MS marker analysis using STRUCTURE and NJ tree indicated that the west Himalayan *A. thaliana* populations were structured. In STRUCTURE analysis, at lower *K*, the accessions grouped at altitudinal level but gradually; at higher *K*, they assembled according to their population affiliation. This finding was corroborated by NJ tree where major branches represented altitudes and external clades represented populations. Only Mun and Dha showed lack of population-level structure. On account of the geographical and altitudinal proximity of this pair of populations, migration might play a key role in determining their inter-population variability, although in PCoA analysis, population-level clusters could not be detected; but clustering was clearly evident at altitudinal level. For a highly selfing plant species, the structured distribution of genetic variation is consistent with *A. thaliana* and has been reported previously (Todokoro *et al*. 1995; Kuittinen *et al*. 2002; Ostrowski *et al*. 2006). On the other hand, the absence of population structure in earlier studies has been interpreted as the result of human disturbance (King *et al*. 1993; Miyashita *et al*. 1999; Vander Zwan *et al*. 2000; Maloof *et al*. 2001). But the present populations were mostly from the undisturbed areas except Deh, which is otherwise geographically isolated from the rest. The observation of significantly positive correlations with geographical distances in these populations further supported the evidence of IBD in the west Himalayan region. These results are consistent with similar analyses of genetic structure both at regional level (Berge *et al*. 1998; Pico *et al*. 2008) and at global level (Nordborg *et al*. 2005; Ostrowski *et al*. 2006; Schmid *et al*. 2006; Beck *et al*. 2008). The low Ho when compared with He indicates a high inbreeding rate, a common phenomenon in highly selfing species like *A. thaliana* (Platt *et al*. 2010). Such patterns of heterozygosities and population structures have been previously observed in *A. thaliana* populations using different markers (Abbott and Gomes 1989; Stenøien *et al*. 2005; Pico *et al*. 2008; Bomblies *et al*. 2010).

The genetic clustering observed in case of MS markers was absent with CP loci due to rarity of PICs in them. Yin *et al*. (2010) have also encountered low nucleotide diversity in Chinese populations resulting in a poorly resolved tree. The CP genome is known to have low nucleotide substitution rate when compared with the nuclear genome (Wolfe *et al*. 1987; Smith 2015). Several studies have reported poor phylogenetic resolution at species and lower taxonomic levels due to small substitution rates in plants (Lee *et al*. 2005; Roy *et al*. 2010). Although the nucleotide diversity of Himalayan accessions was much higher than that of Yangtze River, still they were not high enough to achieve significant clustering.

### West Himalayan populations with respect to world populations

The six populations analysed using CP loci contained a substantial amount of genetic variation, since all of them included several haplotypes. However, caution must be taken in interpreting these data since genetic diversity of local populations might depend on population size, age and habitats as well as types of markers used for diversity estimation (Pico *et al*. 2008). The west Himalayan populations analysed here were from diverse habitat types and mostly growing in natural sites. Such populations may contain large genetic variations when compared with smaller or younger populations. But our study may be limited due to the small number of populations. It is worth mentioning here that despite all of our efforts, we could not locate more populations in this rugged mountainous region.

Phylogenetic tree analysis using CP markers showed no geographical affiliation in clustering of the rest of the world accessions, but accessions from Yangtze River and west Himalaya (excluding Kas-1) formed one group distinct from the rest of the world accessions. This polytomous clustering was primarily attributable to lack of PIC's. The two strongly supported branches in the NJ tree were obtained as a result of the use of indels as phylogenetic information. Several other studies involving regional (Sharbel *et al*. 2000; Jørgensen and Mauricio 2004; Stenøien *et al*. 2005) as well as global (Bergelson *et al*. 1998; Miyashita *et al*. 1999; Erschadi *et al*. 2000; Barth *et al*. 2002; Bakker *et al*. 2006) accessions have reported little or no clustering of accessions based on their geographical location on the basis of different markers, whereas clustering on the basis of geographical location has also been reported (Sharbel *et al*. 2000; Lundemo *et al*. 2009). This pattern of neutral genetic variability has been explained by rapid spread followed by little gene flow among populations of *A. thaliana*. The inference of rapid expansion of these populations is further strengthened by the results of neutral mutation hypothesis test where most of the CP loci were found to have significantly negative Tajima’s *D*, FL-D and FL-F. A significantly negative Tajima's *D* value is a general feature of the *A. thaliana* genome (Schmid *et al*. 2005) and is consistent with demographic forces, such as population growth, rather than non-neutral forces such as selection (Beck *et al*. 2008).

The results of haplotype analysis further corroborate the genetic distinction between these two groups. As observed by Yin *et al*. (2010), this study also indicated the presence of two major haplogroups in the combined data set. However, we found one haplogroup consisted entirely of populations from the west Himalaya and Yangtze River and the other consisted of the rest of the world populations. Inclusion of the west Himalayan populations clearly indicated a dimorphism between the rest of the world populations and west Himalaya–Yangtze River populations, in contrast to what was observed in the Yin *et al*. (2010) study. Although this dimorphism was supported by only two nucleotide character states, high statistical branch support achieved in the phylogenetic analysis further substantiates the presence of these two worldwide groups. It was notable that the cpDNA diversity of Yangtze River accessions was the lowest among all the regions (0.00017). This was further validated by the low haplotype diversity of Yangtze River populations. Ancestral haplotype analysis showed one of the high-frequency haplotypes (CHN-AHyxx) as the ancestral haplotype of Haplogroup I and this haplotype also notably included the Kas-2 accession from the west Himalaya. This suggests that the Yangtze River populations might have arisen from the west Himalaya population but experienced founder effect phenomena leading to its near complete homogenization in cpDNA diversity. Our results further clarified the doubt about the origin of Kas-1, which was not clustered with accessions of west Himalaya or Yangtze River accessions. Earlier studies have had raised doubts about the origin of Kas-1 and they were proposed to include more accessions from this region to clarify its origin more conclusively (Vander Zwan *et al*. 2000; Yin *et al*. 2010).

### In isolation evolution of west Himalayan populations

The divergence time of the west Himalaya population was about 0.45 mya. This comparatively long demographic history and the existing geographical and environmental barriers of the Himalaya might have aided in isolation evolution of these populations. Further, the common ancestor of the west Himalayan and Yangtze River populations might have split from the rest of the world, most probably from the central Asian region at 0.49 mya. *Arabidopsis thaliana* collected from the west Himalayan region occupied a broad diversity of habitats with varied climatic conditions that can be primarily attributed to the steep altitudinal gradient. These diverse habitats together with large genetic variations observed within the west Himalayan populations also suggest a longer demographic history of *A. thaliana* in this region. This was further supported by the results of mismatch distribution analyses. The results suggest that the initial time of expansion of west Himalayan populations to be about 130 000 years, whereas population expansion in Yangtze River began at about 84 000 years ago, which is slightly lower than the estimation of Yin *et al*. (2010). The results of haplotype and divergence time analyses of the west Himalaya and Yangtze populations ruled out any recent colonization of west Himalayan populations. Our results further suggest in-isolation evolution of west Himalayan populations and might have served as a source for eastward expansions as this region contains an amount of neutral genetic diversity similar to that of the rest of the world. The decrease in genetic diversity in the Yangtze River indicates west–east expansions. We hypothesize that besides being the source of European populations as suggested by Sharbel *et al*. (2000), central Asian populations might be the source of west Himalayan populations as well, which further expanded eastwards. High genetic diversity of west Himalayan populations suggests this region as a possible centre of diversity and source of worldwide populations, but extensive sampling of large number of populations may be required to prove this hypothesis. Further, analysis including collections from the east Himalaya, eastern China and Korean peninsula may provide further insight to the west–east expansion model. However, as indicated by other studies, populations of Japan may not be part of this natural expansion due to the existing physical barrier (Sea of Japan) rather than a recent colonization (Todokoro *et al*. 1995).

## Conclusions

The six west Himalayan *A. thaliana* populations analysed in this study were highly diverse and structured, even at altitudinal level. Although we compared the west Himalayan populations with diverse collections of worldwide accessions, the genetic diversity indices were comparable with the world populations. It is clear from this study that these populations are genetically distinct from the rest of the world populations. West Himalayan populations are not recently colonized as indicated by time of divergence estimate and demographic expansion model test. The geophysical nature of this region might have played a major role in shaping the population structure in this region. This region might be the source of west–east expansion model, but further studies are needed to corroborate the hypothesis.

## Accession Numbers

GenBank Accession Numbers: KP191115–KP191466 and KT366267–KT366442.

## Sources of Funding

The study was supported by Council of Scientific and Industrial Research (CSIR), New Delhi, Project: SIMPLE (BSC-109).

## Contributions by the Authors

S.R. designed and conceptualized the study. A.T., S.S., P.M., A.S., A.M.T. and S.N.J. conducted the experiments. S.R. and A.T. wrote the manuscript.

## Conflict of Interest Statement

None declared.

## Supporting Information

The following additional information is available in the online version of this article –

**Table S1.** Detailed geographical locations of sample collection sites and their habitats.

**Table S2.** Details of 19 MS loci data.

**Table S3.** Pairwise genetic distances (*F*_ST_) between west Himalayan populations. All the values are highly significant (*P* < 0.0001).

**Table S4.** Pairwise geographical distances between west Himalayan populations (in km).

**Table S5.** Results of haplotype analysis of west Himalayan (WH) populations and the rest of the world (RW) accessions using CP markers. Showing outgroup weights (in decreasing order) of the haplotypes and the corresponding accessions in the haplotype.

**Table S6.** Nucleotide diversity (*π*) and the results of neutral mutation hypothesis tests for the 11 CP marker data sets of WH populations. ns, not significant (*P* > 0.05); s, significant (*P* < 0.05); vs, very significant (*P* < 0.01).

**Table S7.** Results of mismatch distribution analysis using CP markers of west Himalaya (WH) and Yangtze River (YR) populations.

**Table S8.** Divergence time estimation. Mean divergence time in million years ago of outgroup species (calibration time points), rest of world (RW), west Himalaya (WH), west Himalaya + Yangtze River (HY) and Yangtze river (YR). Also included is 95% upper and lower higher probability density (HPD) and effective sample size (ESS).

**Figure S1.** Mean probability (*D*) of ln(*K*).

**Figure S2.** Consensus NJ tree of 1000 bootstrap replicates for WH accessions (total 48) constructed using concatenated sequences of 11 CP markers. The tree was constructed employing indel coding method MCIC. Figures on the nodes represent bootstrap branch support.

**Figure S3.** Percentage contributions to the genetic variability of the co-ordinates calculated using principal co-ordinate analysis of west Himalayan samples on the basis of MS markers. *x*-axis = co-ordinate, *y*-axis = percentage contribution of the corresponding co-ordinate to the overall variability.

Additional Information

## References

[PLV145C1] Abbott RJ, Gomes MF 1989 Population genetic structure and outcrossing rate of *Arabidopsis thaliana* (L.) Heynh. Heredity 62:411–418. 10.1038/hdy.1989.56

[PLV145C2] Bakker EG, Stahl EA, Toomajian C, Nordborg M, Kreitman M, Bergelson J 2006 Distribution of genetic variation within and among local populations of *Arabidopsis thaliana* over its species range. Molecular Ecology 15:1405–1418. 10.1111/j.1365-294X.2006.02884.x 16626462

[PLV145C3] Barth S, Melchinger AE, Lübberstedt TH 2002 Genetic diversity in *Arabidopsis thaliana* L. Heynh. investigated by cleaved amplified polymorphic sequence (CAPS) and inter-simple sequence repeat (ISSR) markers. Molecular Ecology 11:495–505. 10.1046/j.0962-1083.2002.01466.x 11918784

[PLV145C4] Beck JB, Schmuths H, Schaal BA 2008 Native range genetic variation in *Arabidopsis thaliana* is strongly geographically structured and reflects Pleistocene glacial dynamics. Molecular Ecology 17:902–915. 10.1111/j.1365-294X.2007.03615.x 18179426

[PLV145C5] Bell CJ, Ecker JR 1994 Assignment of 30 microsatellite loci to the linkage map of *Arabidopsis*. Genomics 19:137–144. 10.1006/geno.1994.1023 8188214

[PLV145C6] Berge G, Nordal I, Hestmark G 1998 The effect of breeding systems and pollination vectors on the genetic variation of small plant populations within an agricultural landscape. Oikos 81:17–29. 10.2307/3546463

[PLV145C7] Bergelson J, Stahl E, Dudek S, Kreitman M 1998 Genetic variation within and among populations of *Arabidopsis thaliana*. Genetics 148:1311–1323. 9539444 10.1093/genetics/148.3.1311 PMC1460032

[PLV145C8] Bomblies K, Yant L, Laitinen RA, Kim ST, Hollister JD, Warthmann N, Fitz J, Weigel D 2010 Local-scale patterns of genetic variability, outcrossing, and spatial structure in natural stands of *Arabidopsis thaliana*. PLoS Genetics 6:e1000890 10.1371/journal.pgen.1000890 20361058 PMC2845663

[PLV145C9] Brennan AC, Méndez-Vigo B, Haddioui A, Martínez-Zapater JM, Picó FX, Alonso-Blanco C 2014 The genetic structure of *Arabidopsis thaliana* in the south-western Mediterranean range reveals a shared history between North Africa and southern Europe. BMC Plant Biology 14:17 10.1186/1471-2229-14-17 24411008 PMC3890648

[PLV145C10] Breyne P, Rombaut D, Van Gysel A, Van Montagu M, Gerats T 1999 AFLP analysis of genetic diversity within and between *Arabidopsis thaliana* ecotypes. Molecular and General Genetics 261:627–634. 10.1007/s004380050005 10394899

[PLV145C11] Chatterjee D 1939 Studies on the endemic flora of India and Burma. Journal of Royal Asiatic Society of Bengal (Science) 5:19–67.

[PLV145C12] Clement M, Posada D, Crandall KA 2000 TCS: a computer program to estimate gene genealogies. Molecular Ecology 9:1657–1659. 10.1046/j.1365-294x.2000.01020.x 11050560

[PLV145C13] Crayn DM, Quinn CJ 2000 The evolution of the atpβ-rbcL intergenic spacer in the epacrids (Ericales) and its systematic and evolutionary implications. Molecular Phylogenetics and Evolution 16:238–252. 10.1006/mpev.2000.0794 10942610

[PLV145C14] Drummond AJ, Suchard MA, Xie D, Rambaut A 2012 Bayesian phylogenetics with BEAUti and the BEAST 1.7. Molecular Biology and Evolution 29:1969–1973. 10.1093/molbev/mss075 22367748 PMC3408070

[PLV145C15] Edgar RC 2004 MUSCLE: multiple sequence alignment with high accuracy and high throughput. Nucleic Acids Research 32:1792–1797. 10.1093/nar/gkh340 15034147 PMC390337

[PLV145C16] Egan AN, Crandall KA 2008 Incorporating gaps as phylogenetic characters across eight DNA regions: ramifications for North American Psoraleeae (Leguminosae). Molecular Phylogenetics and Evolution 46:532–546. 10.1016/j.ympev.2007.10.006 18039582

[PLV145C17] Erschadi S, Haberer G, Schöniger M, Torres-Ruiz RA 2000 Estimating genetic diversity of *Arabidopsis thaliana* ecotypes with amplified fragment length polymorphisms (AFLP). Theoretical and Applied Genetics 100:633–640.

[PLV145C18] Ersts P 2011 Geographic distance matrix generator (version 1.2.3). American Museum of Natural History, Center for Biodiversity and Conservation http://biodiversityinformatics.amnh.org/open_source/gdmg.

[PLV145C19] Excoffier L, Lischer HEL 2010 Arlequin suite ver 3.5: a new series of programs to perform population genetics analyses under Linux and Windows. Molecular Ecology Resources 10:564–567. 10.1111/j.1755-0998.2010.02847.x 21565059

[PLV145C20] Felsenstein J 2005 PHYLIP (Phylogeny Inference Package) version 3.6. Distributed by the author Seattle: Department of Genome Sciences, University of Washington.

[PLV145C21] Fu YX, Li WH 1993 Statistical tests of neutrality of mutations. Genetics 133:693–709. 8454210 10.1093/genetics/133.3.693 PMC1205353

[PLV145C22] Futuyama DJ 1998 Evolutionary biology. Sunderland: Sinauer.

[PLV145C23] Goudet J 1995 FSTAT (version 1.2): a computer program to calculate F-statistics. Journal of Heredity 86:485–486.

[PLV145C24] Gugerli F 1998 Effect of elevation on sexual reproduction in alpine populations of *Saxifraga oppositifolia* (Saxifragaceae). Oecologia 114:60–66. 10.1007/s004420050420 28307558

[PLV145C25] Haider S, Kueffer C, Edwards PJ, Alexander JM 2012 Genetically based differentiation in growth of multiple non-native plant species along a steep environmental gradient. Oecologia 170:89–99. 10.1007/s00442-012-2291-2 22434406

[PLV145C26] Hamrick JL, Godt MJW 1996 Effects of life history traits on genetic diversity in plant species. Philosophical Transactions of the Royal Society B: Biological Sciences 351:1291–1298. 10.1098/rstb.1996.0112

[PLV145C27] Hanfstingl U, Berry A, Kellogg EA, Costa JT III, Rüdiger W, Ausubel FM 1994 Haplotypic divergence coupled with lack of diversity at the *Arabidopsis thaliana* alcohol dehydrogenase locus: roles for both balancing and directional selection? Genetics 138:811–828. 7851777 10.1093/genetics/138.3.811 PMC1206230

[PLV145C28] He F, Kang D, Ren Y, Qu L-J, Zhen Y, Gu H 2007 Genetic diversity of the natural populations of *Arabidopsis thaliana* in China. Heredity 99:423–431. 10.1038/sj.hdy.6801020 17593944

[PLV145C29] Hoffmann MH 2002 Biogeography of *Arabidopsis thaliana* (l.) heynh. (Brassicaceae). Journal of Biogeography 29:125–134. 10.1046/j.1365-2699.2002.00647.x

[PLV145C30] Hooker JD 1875 *Flora of British**India*, Vol. I London: Reeve.

[PLV145C31] Horton MW, Hancock AM, Huang YS, Toomajian C, Atwell S, Auton A, Muliyati NW, Platt A, Sperone FG, Vilhjálmsson BJ, Nordborg M, Borevitz JO, Bergelson J 2012 Genome-wide patterns of genetic variation in worldwide *Arabidopsis thaliana* accessions from the RegMap panel. Nature Genetics 44:212–216. 10.1038/ng.1042 22231484 PMC3267885

[PLV145C32] Ingvarsson PK, Ribstein S, Taylor DR 2003 Molecular evolution of insertions and deletion in the chloroplast genome of silene. Molecular Biology and Evolution 20:1737–1740. 10.1093/molbev/msg163 12832644

[PLV145C33] Jakobsson M, Hagenblad J, Tavaré S, Säll T, Halldén C, Lind-Halldén C, Nordborg M 2006 A unique recent origin of the allotetraploid species *Arabidopsis suecica*: evidence from nuclear DNA markers. Molecular Biology and Evolution 23:1217–1231. 10.1093/molbev/msk006 16549398

[PLV145C34] Jørgensen S, Mauricio R 2004 Neutral genetic variation among wild North American populations of the weedy plant *Arabidopsis thaliana* is not geographically structured. Molecular Ecology 13:3403–3413. 10.1111/j.1365-294X.2004.02329.x 15487999

[PLV145C35] Joshi AK, Joshi P 2011 Elevational reduction of plant species diversity in high altitudes of Garhwal Himalaya, India. Current Science 100:833.

[PLV145C36] Kalinowski ST 2005 hp-rare 1.0: a computer program for performing rarefaction on measures of allelic richness. Molecular Ecology Notes 5:187–189. 10.1111/j.1471-8286.2004.00845.x

[PLV145C37] King G, Nienhuis J, Hussey C 1993 Genetic similarity among ecotypes of *Arabidopsis thaliana* estimated by analysis of restriction fragment length polymorphisms. Theoretical and Applied Genetics 86:1028–1032. 24194013 10.1007/BF00211057

[PLV145C38] Koch M, Haubold B, Mitchell-Olds T 2001 Molecular systematics of the Brassicaceae: evidence from coding plastidic matK and nuclear Chs sequences. American Journal of Botany 88:534–544. 10.2307/2657117 11250830

[PLV145C39] Koch MA, Haubold B, Mitchell-Olds T 2000 Comparative evolutionary analysis of chalcone synthase and alcohol dehydrogenase loci in *Arabidopsis*, Arabis, and related genera (Brassicaceae). Molecular Biology and Evolution 17:1483–1498. 10.1093/oxfordjournals.molbev.a026248 11018155

[PLV145C40] Kuittinen H, Salguero D, Aguade M 2002 Parallel patterns of sequence variation within and between populations at three loci of *Arabidopsis thaliana*. Molecular Biology and Evolution 19:2030–2034. 10.1093/oxfordjournals.molbev.a004027 12411612

[PLV145C41] Le Corre V 2005 Variation at two flowering time genes within and among populations of *Arabidopsis thaliana*: comparison with markers and traits. Molecular Ecology 14:4181–4192. 10.1111/j.1365-294X.2005.02722.x 16262868

[PLV145C42] Lee C, Kim SC, Lundy K, Santos-Guerra A 2005 Chloroplast DNA phylogeny of the woody *Sonchus* alliance (Asteraceae: Sonchinae) in the Macaronesian Islands. American Journal of Botany 92:2072–2085. 10.3732/ajb.92.12.2072 21646124

[PLV145C43] Lundemo S, Falahati-Anbaran M, Stenøien HK 2009 Seed banks cause elevated generation times and effective population sizes of *Arabidopsis thaliana* in northern Europe. Molecular Ecology 18:2798–2811. 10.1111/j.1365-294X.2009.04236.x 19500249

[PLV145C44] Maloof JN, Borevitz JO, Dabi T, Lutes J, Nehring RB, Redfern JL, Trainer GT, Wilson JM, Asami T, Berry CC, Weigel D, Chory J 2001 Natural variation in light sensitivity of *Arabidopsis*. Nature Genetics 29:441–446. 10.1038/ng777 11726931

[PLV145C45] Mitchell-Olds T 2001 *Arabidopsis thaliana* and its wild relatives: a model system for ecology and evolution. Trends in Ecology and Evolution 16:693–700. 10.1016/S0169-5347(01)02291-1

[PLV145C46] Mitchell-Olds T, Schmitt J 2006 Genetic mechanisms and evolutionary significance of natural variation in *Arabidopsis*. Nature 441:947–952. 10.1038/nature04878 16791187

[PLV145C47] Miyashita NT, Kawabe A, Innan H 1999 DNA variation in the wild plant *Arabidopsis thaliana* revealed by amplified fragment length polymorphism analysis. Genetics 152:1723–1731. 10430596 10.1093/genetics/152.4.1723 PMC1460704

[PLV145C48] Müller K 2005 SeqState: primer design and sequence statistics for phylogenetic DNA datasets. Applied Bioinformatics 4:65–69. 10.2165/00822942-200504010-00008 16000015

[PLV145C49] Müller K 2006 Incorporating information from length-mutational events into phylogenetic analysis. Molecular Phylogenetics and Evolution 38:667–676. 10.1016/j.ympev.2005.07.011 16129628

[PLV145C50] Nayar MP 1996 “Hot spots” of endemic plants of India, Nepal and Bhutan. Thiruvananthapuram: Tropical Botanic Garden and Research Institute.

[PLV145C51] Nordborg M, Hu TT, Ishino Y, Jhaveri J, Toomajian C, Zheng H, Bakker E, Calabrese P, Gladstone J, Goyal R, Jakobsson M, Kim S, Morozov Y, Padhukasahasram B, Plagnol V, Rosenberg NA, Shah C, Wall JD, Wang J, Zhao K, Kalbfleisch T, Schulz V, Kreitman M, Bergelson J 2005 The pattern of polymorphism in *Arabidopsis thaliana*. PLoS Biology 3:e196 10.1371/journal.pbio.0030196 15907155 PMC1135296

[PLV145C52] Ohsawa T, Ide Y 2008 Global patterns of genetic variation in plant species along vertical and horizontal gradients on mountains. Global Ecology and Biogeography 17:152–163. 10.1111/j.1466-8238.2007.00357.x

[PLV145C53] Ostrowski M-F, David J, Santoni S, Mckhann H, Reboud X, Le Corre V, Camilleri C, Brunel D, Bouchez D, Faure B, Bataillon T 2006 Evidence for a large-scale population structure among accessions of *Arabidopsis thaliana*: possible causes and consequences for the distribution of linkage disequilibrium. Molecular Ecology 15:1507–1517. 10.1111/j.1365-294X.2006.02865.x 16629807

[PLV145C54] Pandit MK, Bhaskar A, Kumar V 2000 Floral diversity of Goriganga Valley in the Central Himalayan highlands. Journal of Bombay Natural History Society 97:184–192.

[PLV145C55] Peakall R, Smouse PE 2012 GenAlEx 6.5: genetic analysis in Excel. Population genetic software for teaching and research—an update. Bioinformatics 28:2537–2539. 10.1093/bioinformatics/bts460 22820204 PMC3463245

[PLV145C56] Pico FX, Méndez-Vigo B, Martínez-Zapater JM, Alonso-Blanco C 2008 Natural genetic variation of *Arabidopsis thaliana* is geographically structured in the Iberian Peninsula. Genetics 180:1009–1021. 10.1534/genetics.108.089581 18716334 PMC2567352

[PLV145C57] Pigliucci M 2002 Ecology and evolutionary biology of *Arabidopsis*. Arabidopsis Book 1:e0003 10.1199/tab.0003 22303188 PMC3243336

[PLV145C58] Platt A, Horton M, Huang YS, Li Y, Anastasio AE, Mulyati NW, Agren J, Bossdorf O, Byers D, Donohue K, Dunning M, Holub EB, Hudson A, Le Corre V, Loudet O, Roux F, Warthmann N, Weigel D, Rivero L, Scholl R, Nordborg M, Bergelson J, Borevitz JO 2010 The scale of population structure in *Arabidopsis thaliana*. PLoS Genetics 6:e1000843 10.1371/journal.pgen.1000843 20169178 PMC2820523

[PLV145C59] Pluess AR, Stöcklin J 2005 The importance of population origin and environment on clonal and sexual reproduction in the alpine plant Geum reptans. Functional Ecology 19:228–237. 10.1111/j.0269-8463.2005.00951.x

[PLV145C60] Pritchard JK, Stephens M, Donnelly P 2000 Inference of population structure using multilocus genotype data. Genetics 155:945–959. 10835412 10.1093/genetics/155.2.945 PMC1461096

[PLV145C61] Provan J, Campanella JJ 2003 Patterns of cytoplasmic variation in *Arabidopsis thaliana* (Brassicaceae) revealed by polymorphic chloroplast microsatellites. Systematic Botany 28:578–583.

[PLV145C62] Rosenberg MS, Anderson CD 2011 PASSaGE: pattern analysis, spatial statistics and geographic exegesis. Version 2. Methods in Ecology and Evolution 2:229–232. 10.1111/j.2041-210X.2010.00081.x

[PLV145C63] Roy S, Tyagi A, Shukla V, Kumar A, Singh UM, Chaudhary LB, Datt B, Bag SK, Singh PK, Nair NK, Husain T, Tuli R 2010 Universal plant DNA barcode loci may not work in complex groups: a case study with Indian *Berberis* species. PLoS ONE 5:e13674 10.1371/journal.pone.0013674 21060687 PMC2965122

[PLV145C64] Rozas J, Sanchez-DelBarrio JC, Messeguer X, Rozas R 2003 DnaSP, DNA polymorphism analyses by the coalescent and other methods. Bioinformatics 19:2496–2497. 10.1093/bioinformatics/btg359 14668244

[PLV145C65] Sall T, Jakobsson M, Lind-Halldén C, Halldén C 2003 Chloroplast DNA indicates a single origin of the allotetraploid *Arabidopsis suecica*. Journal of Evolutionary Biology 16:1019–1029. 10.1046/j.1420-9101.2003.00554.x 14635917

[PLV145C66] Schmid KJ, Ramos-Onsins S, Ringys-Beckstein H, Weisshaar B, Mitchell-Olds T 2005 A multilocus sequence survey in *Arabidopsis thaliana* reveals a genome-wide departure from a neutral model of DNA sequence polymorphism. Genetics 169:1601–1615. 10.1534/genetics.104.033795 15654111 PMC1449538

[PLV145C67] Schmid KJ, Törjék O, Meyer R, Schmuths H, Hoffmann MH, Altmann T 2006 Evidence for a large-scale population structure of *Arabidopsis thaliana* from genome-wide single nucleotide polymorphism markers. Theoretical and Applied Genetics 112:1104–1114. 10.1007/s00122-006-0212-7 16453134

[PLV145C68] Schmuths H, Hoffmann M, Bachmann K 2004 Geographic distribution and recombination of genomic fragments on the short arm of chromosome 2 of *Arabidopsis thaliana*. Plant Biology 6:128–139. 10.1055/s-2004-817837 15045663

[PLV145C69] Schuelke M 2000 An economic method for the fluorescent labeling of PCR fragments. Nature Biotechnology 18:233–234. 10.1038/72708 10657137

[PLV145C70] Sharbel TF, Haubold B, Mitchell-olds T 2000 Genetic isolation by distance in *Arabidopsis thaliana*: biogeography and postglacial colonization of Europe. Molecular Ecology 9:2109–2118. 10.1046/j.1365-294X.2000.01122.x 11123622

[PLV145C71] Singh A, Tyagi A, Tripathi AM, Gokhale SM, Singh N, Roy S 2015 Morphological trait variations in the west Himalayan (India) populations of *Arabidopsis thaliana* along altitudinal gradients. Current Science 108:2213.

[PLV145C72] Smith DR 2015 Mutation rates in plastid genomes: they are lower than you might think. Genome Biology and Evolution 7:1227–1234. 10.1093/gbe/evv069 25869380 PMC4453064

[PLV145C73] Stenøien HK, Fenster CB, Tonteri A, Savolainen O 2005 Genetic variability in natural populations of *Arabidopsis thaliana* in northern Europe. Molecular Ecology 14:137–148. 10.1111/j.1365-294X.2004.02359.x 15643957

[PLV145C74] Swofford DL 2003 PAUP*. Phylogenetic Analysis Using Parsimony (* and Other Methods). Version 4. Sunderland, MA: Sinauer Associates.

[PLV145C75] Symonds VV, Lloyd AM 2003 An analysis of microsatellite loci in *Arabidopsis thaliana*: mutational dynamics and application. Genetics 165:1475–1488. 14668396 10.1093/genetics/165.3.1475 PMC1462854

[PLV145C76] Tajima F 1989 Statistical method for testing the neutral mutation hypothesis by DNA polymorphism. Genetics 123:585–595. 2513255 10.1093/genetics/123.3.585 PMC1203831

[PLV145C77] Tamura K, Peterson D, Peterson N, Stecher G, Nei M, Kumar S 2011 MEGA5: molecular evolutionary genetics analysis using maximum likelihood, evolutionary distance, and maximum parsimony methods. Molecular Biology and Evolution 28:2731–2739. 10.1093/molbev/msr121 21546353 PMC3203626

[PLV145C78] Till-Bottraud I, Gaudeul M 2002 Intraspecific genetic diversity in alpine plants. In: Körner C., Spehn EM, eds. Mountain biodiversity: a global assessment. New York: Parthenon Publishing Group.

[PLV145C79] Todokoro S, Terauchi R, Kawano S 1995 Microsatellite polymorphisms in natural populations of *Arabidopsis thaliana* in Japan. The Japanese Journal of Genetics 70:543–554. 10.1266/jjg.70.543

[PLV145C80] Vander Zwan C, Brodie SA, Campanella JJ 2000 The intraspecific phylogenetics of *Arabidopsis thaliana* in worldwide populations. Systematic Botany 25:47–59. 10.2307/2666672

[PLV145C81] Wang YH, Upadhyaya HD, Burrell AM, Sahraeian SM, Klein RR, Klein PE 2013 Genetic structure and linkage disequilibrium in a diverse, representative collection of the C4 model plant, *Sorghum bicolor*. Genes Genomes Genetics (G3) 3:783–793. 23704283 10.1534/g3.112.004861 PMC3656726

[PLV145C82] Weigel D 2012 Natural variation in *Arabidopsis*: from molecular genetics to ecological genomics. Plant Physiology 158:2–22. 10.1104/pp.111.189845 22147517 PMC3252104

[PLV145C83] Wolfe KH, Li WH, Sharp PM 1987 Rates of nucleotide substitution vary greatly among plant mitochondrial, chloroplast, and nuclear DNAs. Proceedings of the National Academy of Sciences of the USA 84:9054–9058. 10.1073/pnas.84.24.9054 3480529 PMC299690

[PLV145C84] Yin P, Kang J, He F, Qu LJ, Gu H 2010 The origin of populations of *Arabidopsis thaliana* in China, based on the chloroplast DNA sequences. BMC Plant Biology 10:22 10.1186/1471-2229-10-22 20141622 PMC2827422

