# Supplementary material for: Genetic diversity and population structure of Arabidopsis thaliana along an altitudinal gradient
Source: AoB Plants. 2015 Dec 15;8:plv145. doi: 10.1093/aobpla/plv145 (PMC4719038; doi:10.1093/aobpla/plv145)
Supplement: Additional Information [file supp_8_plv145_index.html]

Genetic diversity and population structure of Arabidopsis thaliana along an altitudinal gradient — Genetic diversity and population structure of Arabidopsis thaliana along an altitudinal gradient — Additional Information 

# Genetic diversity and population structure of *Arabidopsis thaliana* along an altitudinal gradient

## Additional Information

Additional Information

- Supplementary file1 - doc file
- Supplementary file2 - txt file
- Supplementary file3 - doc file
- Supplementary file4 - ppt file
