# Supplementary material for: Genetic diversity and population structure of Arabidopsis thaliana along an altitudinal gradient
Source: AoB Plants. 2015 Dec 15;8:plv145. doi: 10.1093/aobpla/plv145 (PMC4719038; doi:10.1093/aobpla/plv145)
Supplement: Additional Information [file supp_plv145_plv145supp_file1.doc]

**Table S1**. Detailed geographical locations of sample collection sites and their habitats. Accessions are named by three letters from the name of location followed by numeric to indicate individual number.

| Location | Population | Latitude | Longitude | Altitude in m amsl | Habitat | Accessions |
| --- | --- | --- | --- | --- | --- | --- |
| Chitkul |  | 31° 21' 0.954" | 78° 26' 30.5694" | 3400 (High altitude) | Undisturebed mountanous slope along river valley | Chi-1 |
|  |  |  |  |  |  | Chi-2 |
|  |  |  |  |  |  | Chi-25 |
|  |  |  |  |  |  | Chi-33 |
|  |  |  |  |  |  | Chi-35 |
|  |  |  |  |  |  | Chi-37 |
|  |  |  |  |  |  | Chi-38 |
|  | Chi |  |  |  |  | Chi-4 |
| Dehradun |  | 30° 20' 34.1196" | 77° 59' 57.5694" | 700 (Low altitude) | Highly disturbed lawn | Deh-1 |
|  |  |  |  |  |  | Deh-13 |
|  |  |  |  |  |  | Deh-17 |
|  |  |  |  |  |  | Deh-25 |
|  |  |  |  |  |  | Deh-3 |
|  |  |  |  |  |  | Deh-35 |
|  |  |  |  |  |  | Deh-37 |
|  | Deh |  |  |  |  | Deh-5 |
| Dhapa |  | 30° 6' 53.7588" | 80° 14' 25.1376" | 1800 (Medium altitude) | Undisturbed mountanous slope | Dha-03 |
|  |  |  |  |  |  | Dha-04 |
|  |  |  |  |  |  | Dha-06 |
|  |  |  |  |  |  | Dha-07 |
|  |  |  |  |  |  | Dha-09 |
|  |  |  |  |  |  | Dha-10 |
|  |  |  |  |  |  | Dha-12 |
|  | Dha |  |  |  |  | Dha-15 |
| Koksar |  | 32° 24' 29.9406" | 77° 15' 21.3258" | 3400 (High altitude) | Undisturbed arid river valley | Kok-03 |
|  |  |  |  |  |  | Kok-07 |
|  |  |  |  |  |  | Kok-08 |
|  |  |  |  |  |  | Kok-09 |
|  |  |  |  |  |  | Kok-20 |
|  |  |  |  |  |  | Kok-21 |
|  |  |  |  |  |  | Kok-22 |
|  | Kok |  |  |  |  | Kok-25 |
| Munsiyari |  | 30° 4' 50.451" | 80° 14' 10.4778" | 2000 (Medium altitude) | Undisturbed mountanous slope | Mun-1 |
|  |  |  |  |  |  | Mun-11 |
|  |  |  |  |  |  | Mun-14 |
|  |  |  |  |  |  | Mun-2 |
|  |  |  |  |  |  | Mun-3 |
|  |  |  |  |  |  | Mun-5 |
|  |  |  |  |  |  | Mun-8 |
|  | Mun |  |  |  |  | Mun-9 |
| Sangla |  | 31° 25' 22.1592" | 78° 15' 54.8598" | 2600 (High altitude) | Along undisturbed mountanous slope and river valley | San-1 |
|  |  |  |  |  |  | San-18 |
|  |  |  |  |  |  | San-19 |
|  |  |  |  |  |  | San-2 |
|  |  |  |  |  |  | San-25 |
|  |  |  |  |  |  | San-28 |
|  |  |  |  |  |  | San-29 |
|  |  |  |  |  |  | San-3 |
