# Supplementary material for: Genetic diversity and population structure of Arabidopsis thaliana along an altitudinal gradient
Source: AoB Plants. 2015 Dec 15;8:plv145. doi: 10.1093/aobpla/plv145 (PMC4719038; doi:10.1093/aobpla/plv145)
Supplement: Additional Information [file supp_plv145_plv145supp_file3.doc]

**Table S3.** Pairwise genetic distances (*F*_ST_ ) between West Himalayan populations. All the values are highly significant (P<0.0001)

|  | Chi | Deh | Dha | Kok | Mun | San |
| --- | --- | --- | --- | --- | --- | --- |
|  |  |  |  |  |  |  |
| Chi | 0 |  |  |  |  |  |
|  |  |  |  |  |  |  |
| Deh | 0.59063 | 0 |  |  |  |  |
|  |  |  |  |  |  |  |
| Dha | 0.59014 | 0.49634 | 0 |  |  |  |
|  |  |  |  |  |  |  |
| Kok | 0.423 | 0.4939 | 0.50173 | 0 |  |  |
|  |  |  |  |  |  |  |
| Mun | 0.61515 | 0.49064 | 0.18993 | 0.52886 | 0 |  |
|  |  |  |  |  |  |  |
| San | 0.41805 | 0.49302 | 0.50972 | 0.35402 | 0.52814 | 0 |
|  |  |  |  |  |  |  |

**Table S4.** Pairwise geographical distances between West Himalayan populations (in Km)

|  | Chi | Deh | Dha | Kok | Mun | San |
| --- | --- | --- | --- | --- | --- | --- |
|  |  |  |  |  |  |  |
| Chi | 0 |  |  |  |  |  |
|  |  |  |  |  |  |  |
| Deh | 119.86 | 0 |  |  |  |  |
|  |  |  |  |  |  |  |
| Dha | 220.28 | 217.03 | 0 |  |  |  |
|  |  |  |  |  |  |  |
| Kok | 162.6 | 240.54 | 381.83 | 0 |  |  |
|  |  |  |  |  |  |  |
| Mun | 222.4 | 217.16 | 3.83 | 384.14 | 0 |  |
|  |  |  |  |  |  |  |
| San | 18.62 | 122.88 | 238.5 | 145.36 | 240.57 | 0 |
|  |  |  |  |  |  |  |

**Table S5.** Results of haplotype analysis of West Himalayan (WH) populations and rest of the world (RW) accessions using CP markers. Showing outgroup weights (in decreasing order) of the haplotypes and the corresponding accessions in the haplotype.

| Representative haplotype | Outgroup weight | Accessions in the haplotype |
| --- | --- | --- |
| CZE_Ta_0 | 0.123894 | FRA_Ag_0,JPN_Hiro,POL_Lip_0,RUS_Per2,USA_FM_1_0 |
| CHN_AHyxx | 0.115044 |  |
| CAN_Pog_0 | 0.106195 | FIN_Es_0,URS_Est_0 |
| CHN_AHthx | 0.106195 | CHN_CQbbq,CHN_CQtlx,CHN_GSwex,CHN_GZyjx,CHN_HBhax,CHN_HNzjj,CHN_SXcgx,CHN_SXmix,CHN_ZJjds,IND_Kas_2 |
| GBR_HR14 | 0.070796 | ITA_Sei_0,LTU_Kn_0,SCO_Lc_0,USA_BG_1 |
| LBA_Mt_0 | 0.070796 |  |
| AUT_Gr_3 | 0.061947 |  |
| GER_Aa_0 | 0.053097 | USA_Berk,USA_Col_0 |
| FRA_Gy_0 | 0.026549 | GBR_Ba_1 |
| GBR_For_1 | 0.026549 | IRL_Bur_0 |
| AUT_Pi_0 | 0.022124 | IND_Kas_1,ITA_Ct_1,RUS_Ms_0,RUS_Was |
| CHN_HBwcq | 0.022124 | CHN_HNylx,CHN_JXjgs,CHN_JXnfx,CHN_ZJdys |
| CHN_JSnjs | 0.017699 |  |
| KAZ_9481 | 0.017699 |  |
| CHN_XJalt | 0.00885 | CHN_XJqhx |
| DEN_Al_0 | 0.00885 | USA_HS10 |
| GER_Eil_0 | 0.00885 | NED_Hi_0 |
| KAZ_KZ10 | 0.00885 | RUS_N_1 |
| BEL_Ang_0 | 0.004425 |  |
| CPV_Cvi_0 | 0.004425 |  |
| CZE_Blh_1 | 0.004425 |  |
| ESP_Alc_0 | 0.004425 |  |
| ESP_Can_0 | 0.004425 |  |
| FIN_Te_0 | 0.004425 |  |
| GBR_Abd_0 | 0.004425 |  |
| GBR_Cal_0 | 0.004425 |  |
| GER_Anholt_1 | 0.004425 |  |
| IND_Chi_33 | 0.004425 |  |
| IND_Deh_17 | 0.004425 |  |
| IND_Dha_03 | 0.004425 |  |
| IND_Kok_09 | 0.004425 |  |
| IND_Mun_5 | 0.004425 |  |
| IND_San_2 | 0.004425 |  |
| ITA_Bl_1 | 0.004425 |  |
| ITA_Mr_0 | 0.004425 |  |
| JPN_Tsu_0 | 0.004425 |  |
| LTU_Wil_1 | 0.004425 |  |
| MAR_Ita_0 | 0.004425 |  |
| NOR_Bus_0 | 0.004425 |  |
| POL_La_0 | 0.004425 |  |
| RUS_Chi_0 | 0.004425 |  |
| SUI_Bs_1 | 0.004425 |  |
| SUI_Cha_0 | 0.004425 |  |
| SWE_Ost_0 | 0.004425 |  |
| TJK_Sorbo | 0.004425 |  |
| UKR_Rub_1 | 0.004425 |  |

**Table S6.** Nucleotide diversity (π) and the results of neutral mutation hypothesis tests for the 11 CP markers data sets of WH populations. Ns = not significant (P > 0.05), s = significant (P < 0.05), vs = very significant (P < 0.01)

| Locus | length | pi | tajima's D |  | FL-D |  | FL-F |  |
| --- | --- | --- | --- | --- | --- | --- | --- | --- |
| accD | 648 | 0.00227 | -2.07207 | s | -3.9562 | vs | -3.9562 | vs |
| psaJ | 312 | 0.00255 | -1.41995 | ns | -1.55516 | ns | -1.79372 | ns |
| psbE | 722 | 0.00214 | -2.068 | s | -3.02162 | s | -3.17186 | vs |
| rbcL | 1040 | 0.00387 | -1.90892 | s | -3.26429 | s | -3.23439 | vs |
| rpl-16 | 219 | 0.00045 | -1.66462 | ns | -2.78275 | s | -2.85175 | s |
| rpl-20 | 733 | 0.00107 | -2.70154 | vs | -7.6523 | vs | -6.76037 | vs |
| rpoB | 629 | 0.00211 | -1.89979 | s | -4.55928 | vs | -4.23553 | vs |
| trnG | 393 | 0.00152 | -1.99602 | s | -2.21421 | ns | -2.54682 | s |
| trnL | 657 | 0.00232 | -2.18531 | s | -5.91609 | vs | -5.36716 | vs |
| trnR | 307 | 0.00295 | -1.63353 | ns | -2.44985 | s | -2.56889 | s |
| ycf-3 | 569 | 0.00011 | -1.74517 | ns | -4.01762 | vs | -3.86467 | vs |
| combined | 6229 | 0.00209 | -2.36096 | vs | -6.12476 | vs | -5.2979 | vs |

**Table S7.** Results of mismatch distribution analysis using CP markers of West Himalaya(WH) and Yangtze River (YR) populations

|  | WH | YR |
| --- | --- | --- |
| Mismatch observed mean | 36.791 | 1.926 |
| Mismatch observed variance | 3123.706 | 5.624 |
| Tau | 4.5 | 2.9 |
| Theta0 | 11.979 | 0 |
| Theta1 | 825.1 | 1.938 |
| Sum of Squared deviation: | 0.00653 | 0.157286 |
| P(Sim. Ssd >= Obs. Ssd): | 0.6 | 0.09 |
| Harpending's Raggedness | 0.0044 | 0.5729 |
| index: |  |  |
| P(Sim. Rag. >= Obs. Rag.): | 0.43 | 0.05 |

**Table S8: Divergence time estimation.** Mean divergence time in mya (million years ago) of outgroup species (calibration time points), Rest of World (RW), West Himalaya (WH), West Himalaya+ Yangtze River (HY) and Yangtze river (YR). Also included is 95% upper and lower higher probability density (HPD) and effective sample size (ESS).

| TMRCA | HY | RW | WH | YR | *A. Arenosa* | *O. Cabulica* | *A. Suecica* | *A. thaliana* |
| --- | --- | --- | --- | --- | --- | --- | --- | --- |
| mean | 0.45 | 0.62 | 0.45 | 0.29 | 6.61 | 12.42 | 0.64 | 0.62 |
| 95% HPD lower | 0.35 | 0.49 | 0.35 | 0.2 | 5.94 | 12.17 | 0.51 | 0.49 |
| 95% HPD upper | 0.56 | 0.74 | 0.56 | 0.39 | 7.34 | 12.68 | 0.76 | 0.74 |
| ESS | 3147.95 | 2340.11 | 3167.76 | 6103.63 | 24662.44 | 26517.99 | 2244.14 | 2339.81 |
