# Supplementary material for: Genetic diversity and population structure of Arabidopsis thaliana along an altitudinal gradient
Source: AoB Plants. 2015 Dec 15;8:plv145. doi: 10.1093/aobpla/plv145 (PMC4719038; doi:10.1093/aobpla/plv145)
Supplement: Additional Information [file supp_plv145_plv145supp_file4.ppt]

## Slide 1
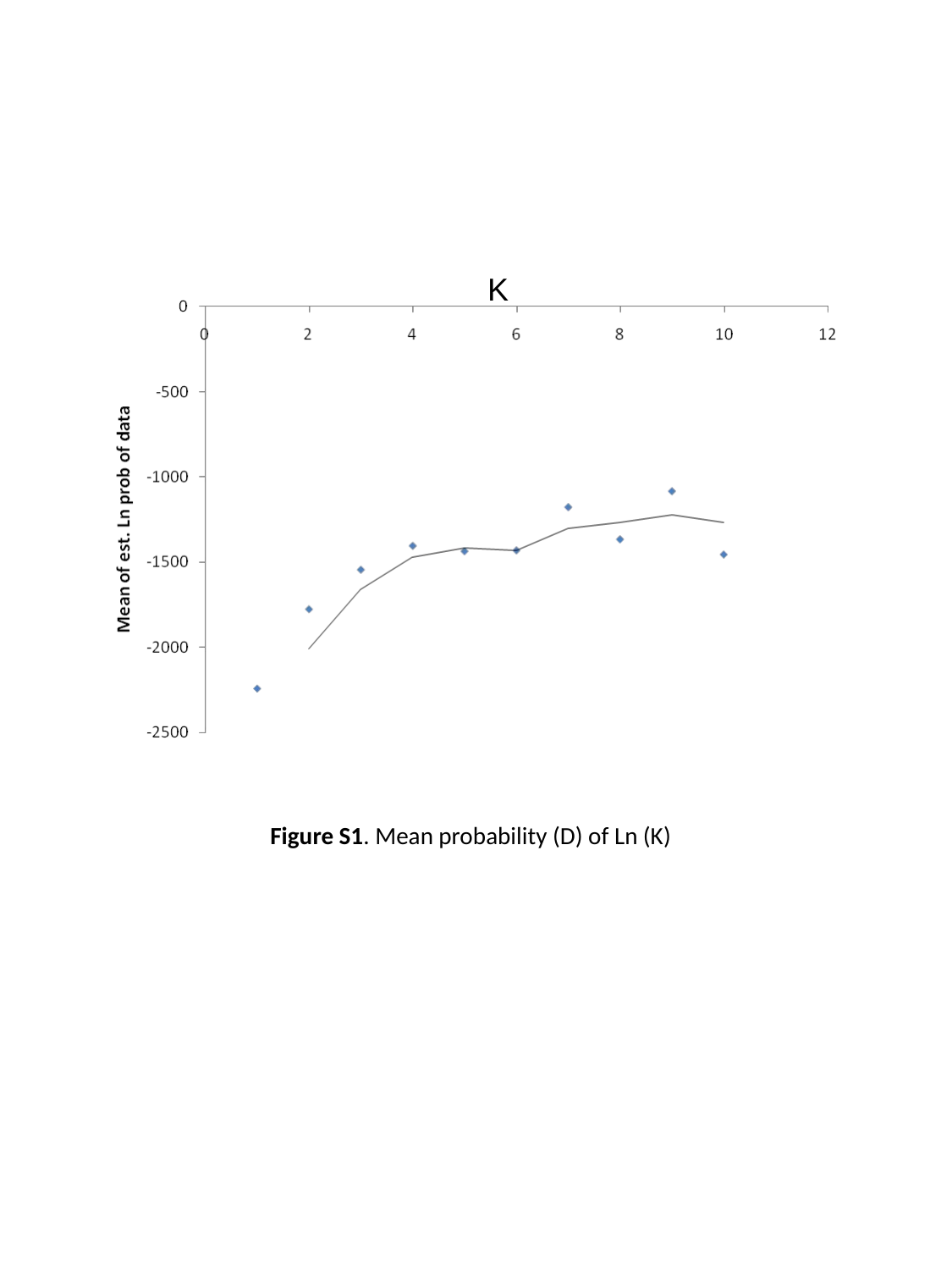

K
Figure S1. Mean probability (D) of Ln (K)

## Slide 2
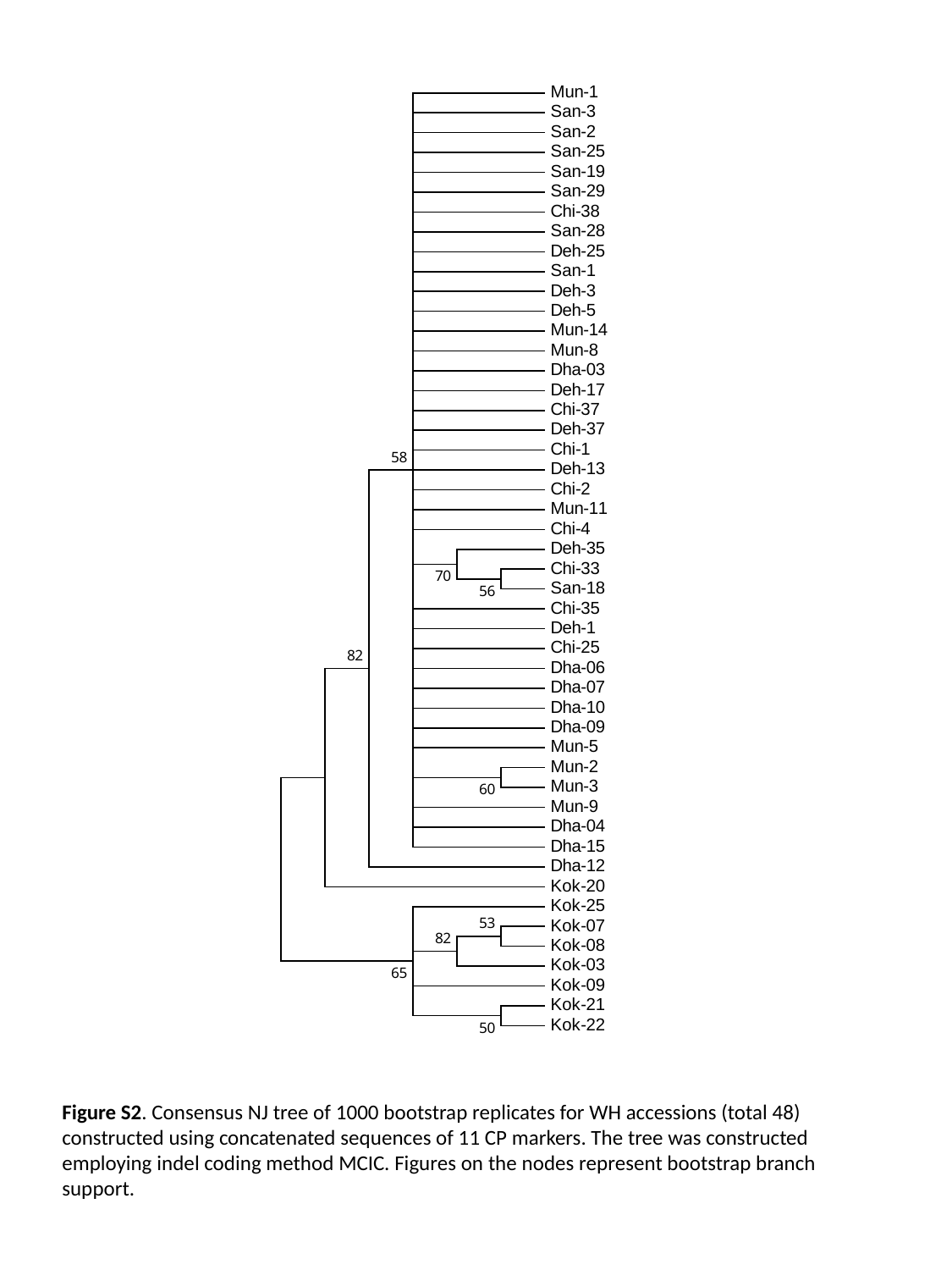

Figure S2. Consensus NJ tree of 1000 bootstrap replicates for WH accessions (total 48) constructed using concatenated sequences of 11 CP markers. The tree was constructed employing indel coding method MCIC. Figures on the nodes represent bootstrap branch support.

## Slide 3
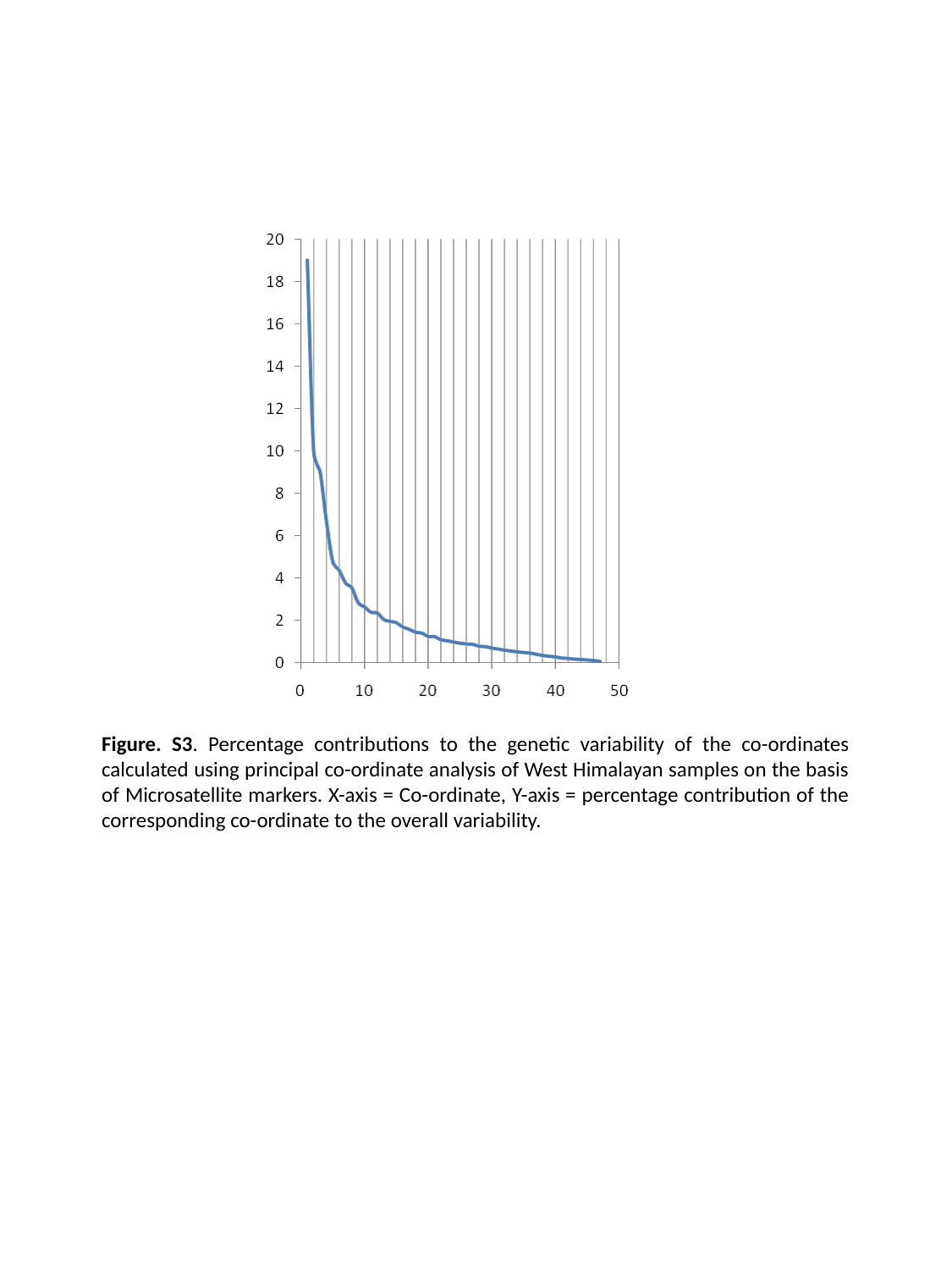

Figure. S3. Percentage contributions to the genetic variability of the co-ordinates calculated using principal co-ordinate analysis of West Himalayan samples on the basis of Microsatellite markers. X-axis = Co-ordinate, Y-axis = percentage contribution of the corresponding co-ordinate to the overall variability.
